# Identifying Breast Cancer-Related Genes Based on a Novel Computational Framework Involving KEGG Pathways and PPI Network Modularity

**DOI:** 10.3389/fgene.2021.596794

**Published:** 2021-08-16

**Authors:** Yan Zhang, Ju Xiang, Liang Tang, Jianming Li, Qingqing Lu, Geng Tian, Bin-Sheng He, Jialiang Yang

**Affiliations:** ^1^School of Computer Science and Engineering, Central South University, Changsha, China; ^2^School of Information Science and Engineering, Changsha Medical University, Changsha, China; ^3^Academician Workstation, Changsha Medical University, Changsha, China; ^4^Neuroscience Research Center & Department of Basic Medical Sciences, Changsha Medical University, Changsha, China; ^5^Qingdao Geneis Institute of Big Data Mining and Precision Medicine, Qingdao, China; ^6^Geneis Beijing Co., Ltd., Beijing, China

**Keywords:** disease-gene prediction, protein-protein interactions, KEGG pathway, breast cancer, network propagation

## Abstract

Complex diseases, such as breast cancer, are often caused by mutations of multiple functional genes. Identifying disease-related genes is a critical and challenging task for unveiling the biological mechanisms behind these diseases. In this study, we develop a novel computational framework to analyze the network properties of the known breast cancer–associated genes, based on which we develop a random-walk-with-restart (RCRWR) algorithm to predict novel disease genes. Specifically, we first curated a set of breast cancer–associated genes from the Genome-Wide Association Studies catalog and Online Mendelian Inheritance in Man database and then studied the distribution of these genes on an integrated protein–protein interaction (PPI) network. We found that the breast cancer–associated genes are significantly closer to each other than random, which confirms the modularity property of disease genes in a PPI network as revealed by previous studies. We then retrieved PPI subnetworks spanning top breast cancer–associated KEGG pathways and found that the distribution of these genes on the subnetworks are non-random, suggesting that these KEGG pathways are activated non-uniformly. Taking advantage of the non-random distribution of breast cancer–associated genes, we developed an improved RCRWR algorithm to predict novel cancer genes, which integrates network reconstruction based on local random walk dynamics and subnetworks spanning KEGG pathways. Compared with the disease gene prediction without using the information from the KEGG pathways, this method has a better prediction performance on inferring breast cancer–associated genes, and the top predicted genes are better enriched on known breast cancer–associated gene ontologies. Finally, we performed a literature search on top predicted novel genes and found that most of them are supported by at least wet-lab experiments on cell lines. In summary, we propose a robust computational framework to prioritize novel breast cancer–associated genes, which could be used for further *in vitro* and *in vivo* experimental validation.

## Introduction

Complex diseases, such as cancers, are often caused by dysfunction of multiple genes. The pathogenic mechanism is often due to molecular abnormalities, which affect the biological function of the body through biomolecular networks, resulting in complex and diverse diseases (Taherian-Fard et al., 2015). The gene families of RAS, MYC, ERBB, and FGFR are common proto-oncogenes (Bi et al., 2018). Although chemoradiotherapy remains the standard treatment for some cancers, the majority of patients, who are sensitive initially, develop resistance after multiple relapses, for example, platinum resistance (Guan and Lu, 2018). Besides this, molecular targeted therapy is expected to be more effective and less toxic compared with chemoradiotherapy. The Food and Drug Administration has approved several targeted medicines. The research and wide application of EGFR-TKI (Tyrosine kinase inhibitors) drugs, mainly including Gefitinib, Erlotinib, Icotinib, Afatinib, Dasatinib, and Osimertinib, have greatly improved the overall survival of patients with lung cancer with the *EGFR* gene mutation. In this case, molecular targeted therapy has brought us much closer to personalized therapy, which will improve the therapeutic effect and prognosis for patients (Colli et al., 2017). Therefore, identifying disease-related genes is a critical and challenging task for the study of complex diseases, which can help us understand the mechanisms of diseases, identify treatment targets, and develop novel treatment strategies (Aitman, 2002; Gill et al., 2014).

Traditional approaches to identification of disease-related genes, such as linkage analysis, involves a candidate list consisting of hundreds of genes, requiring a lot of cost and time for in-depth validation (Gill et al., 2014; Opap and Mulder, 2017). As such, disease-gene prediction has attracted much attention in past decades, and many computational algorithms have been developed to predict disease-related genes to minimize the cost and time for the study of disease-related genes (Chen et al., 2014; Gill et al., 2014; Opap and Mulder, 2017; Luo et al., 2019a,b). Many studies show that genes associated with the same or similar diseases often are more similar in function than others (Goh et al., 2007). Functional similar genes as well as their products often have physical interactions or functional associations. At present, with the rapid development of high-throughput technology, a large number of physical and functional relationships between biomolecules have been revealed, and these form complex biomolecular networks, e.g., protein–protein interaction (PPI) networks (Keshava Prasad et al., 2009), gene co-expression networks, and pathway networks (Kanehisa and Goto, 2000). It is found that a gene is more likely to be related to a disease if there exists direct physical interactions or strong functional associations between it and known disease-related genes. Therefore, “guilt by association” becomes a popular strategy for disease-gene prediction (Oliver, 2000; Wu et al., 2008; Hu et al., 2018), and network propagation, such as random walk, has become a widely used approach for disease-gene prediction (Cowen et al., 2017). However, the existing PPI network is still incomplete, and there is a lot of data noise. How to improve the PPI network so as to enhance the ability to predict disease genes is still a problem that needs further study.

Breast cancer is one of the common malignant tumors among women all over the world. Surgery is still the preferred treatment for breast cancer. However, patients with poor systemic conditions, such as serious diseases in the main organs, are prohibited from using surgical treatment. Therefore, to expand the benefit population and improve the treatment effect of breast cancer patients, targeted therapy occupies the most important position in the treatment of breast cancer (Valencia et al., 2017). To identify breast cancer–related genes more effectively, we conduct analysis and prediction of breast cancer–related genes based on the PPI network and KEGG pathway because PPIs are proven to be very useful in disease-gene prediction, and the physical and functional relationships between genes in the KEGG pathways are stronger and more reliable than others. After collecting disease-gene associations for breast cancer as well as many other diseases, PPIs and KEGG pathway data, we first analyze breast cancer–related genes from two aspects: network and enrichment analysis. Then, to enhance the ability for disease-gene prediction, we propose an improved algorithm (RCRWR), which consists of network reconstruction based on local random walk dynamics and random walk with restart. Further, we also improve the prediction ability for disease-related genes by integrating KEGG pathway data. Finally, we conduct extensive analysis for candidate genes.

The rest of the paper is organized as follows. Section Materials and Methods describes the materials and methods used in the study, including the improved algorithm (RCRWR) for disease-gene prediction. Section Results conducts the analysis of disease-related genes by network and enrichment analysis and then evaluates the performance of RCRWR when predicting genes related to breast cancer and other diseases. The results confirm the effectiveness of RCRWR and the important roles of KEGG pathway data in enhancing the ability of disease-gene prediction. Finally, Section Conclusion draws conclusions.

## Materials and Methods

Here, we first prepare the following data sets: known disease-gene associations, PPIs, and KEGG pathway data. Then, we introduce the methods for statistics of breast cancer-related genes and the improved algorithm for predicting disease-related genes.

### Data SOURCES

#### Disease-Gene Associations

The disease/trait associated genes were retrieved from the National Institutes of Health Genome-Wide Association Studies (GWAS) catalog (https://www.ebi.ac.uk/gwas/) (Danielle et al., 2013) and Online Mendelian Inheritance in Man (OMIM) (https://omim.org/) (Hamosh, 2004). Some GWAS catalog disease categories are closely related but named differently by different investigators, some of which have many overlapping genes (e.g., see Supplementary Tables 1, 2). It is helpful to merge the related groups of diseases. For that purpose, a hierarchical clustering of diseases is applied to cluster these diseases according to their common disease-related genes. Similar diseases in GWAS and OMIM are manually merged based on disease names. The data set was obtained from the previous study (Yang et al., 2016).

#### PPIs

In the various types of data that have been used for the prediction of disease genes, PPIs are the most widely used data. The PPI network was obtained from the database of STRING (https://string-db.org) (von Mering et al., 2003), which quantitatively incorporates several studies and interaction types. In this study, we consider only the undirected and weighted network.

#### KEGG Pathways

We downloaded the KEGG pathway data set from KEGG (Kanehisa and Goto, 2000) (https://www.genome.jp/) and MSigDB (https://www.gsea-msigdb.org) (Liberzon et al., 2011). The KEGG pathway database is a collection of manually drawn pathway maps representing our knowledge on the molecular interaction, reaction, and relation networks for metabolism, genetic information processing, environmental information processing, cellular processes, organismal systems, human diseases, and drug development. MSigDB provides gene sets of canonical KEGG pathways derived from the KEGG pathway database. This data set contains 5,267 unique genes.

Data preparation: We prepare the disease-gene associations, PPI network, and pathway data. Analysis of breast cancer-related genes: We conduct two types of analysis for disease-related genes (network and enrichment). Prediction of breast cancer-related genes: We evaluate the prediction performance based on the PPI network and PPI & KEGG pathway, and then we prioritize the candidate genes related to breast cancer by using all known disease-related genes as a training set. Analysis of candidate genes for breast cancer: We conduct three types of analysis for the candidate genes related to breast cancer (enrichment analysis of GO and KEGG as well as literature validation).

### Statistics of Breast Cancer–Related Genes

#### Network Analysis

First, we extract the disease-gene subnetwork related to a specific disease by retaining genes related to this disease and removing all other genes from the PPI network. We calculate six statistical measures of the network to evaluate the disease-gene subnetwork: (a) the number of genes; (b) the number of edges; (c) the average degrees of nodes; (d) clustering coefficient in the subnetwork; (e) link density, which is defined as ratio of the number of existing interactions to its maximum of possible edges; and (f) a *p*-value is given to evaluate the significance of interaction enrichment in the subnetwork.

Then, we analyze the distribution of breast cancer–related genes in KEGG pathways (e.g., gastric cancer, cellular senescence, human T cell leukemia virus 1 infection, breast cancer, melanoma) by calculating (a) the number of common genes between the pathway and the breast cancer–related gene set; (b) the number of genes in KEGG pathway; (c) the number of edges in the subnetwork of the KEGG pathway; (d) the average degrees of nodes; (e) the clustering coefficient in the subnetwork; and (f) the link density, which is defined as ratio of the number of existing interactions to its maximum of possible edges as well as (g) a *p*-value indicating the significance of gene enrichment in the KEGG pathway.

To demonstrate the higher connectivity of the related subnetworks, we compare these statistical quantities to those of random subsets of genes mapped on the PPI network with the same number of genes and same degree distribution.

#### Enrichment Analysis

Enrichment analysis is a widely used approach to identify biological themes. We analyze the enrichment of the gene set in GO and the pathway. *P*-values using the hypergeometric distribution are defined as

(1) $$p=1-\sum_{i=0}^{k-1}\frac{\left(\begin{array}{c} M\\ i \end{array}\right)\left(\begin{array}{c} N-M\\ n-i \end{array}\right)}{\left(\begin{array}{c} N\\ n \end{array}\right)},$$

where *N* is the total number of genes in the background distribution, *M* is the number of genes with given annotations in that distribution, *n* is the size of the list of genes of interest, and *k* is the number of genes with the annotations in this list. *P*-values are adjusted for multiple comparisons, and *q*-values are also calculated for FDR control.

The clusterProfiler package was used to perform the enrichment analysis for GO terms and KEGG pathways (Yu et al., 2012). As such, the background genes are dependent on the databases used by this package. This package depends on the bioconductor annotation data GO.db and KEGG.db to obtain the maps of the entire GO and KEGG corpus. It provides functions, enrichGO and enrichKEGG, to perform the enrichment test for GO terms and KEGG pathways based on hypergeometric distribution. According to the description of clusterProfiler, the background genes should be all genes within a given annotation file, e.g., the GO annotation file. However, the version of the specific annotation file is dependent on the clusterProfiler package.

### Improved Algorithm for Predicting Breast Cancer-Related Genes

As shown in **Figure 2**, breast cancer–related genes tend to be connected with each other in the PPI network. As such, the network-based algorithms can often provide useful insight to infer breast cancer–related (candidate) genes. In this case, the PPI network is critical. Despite the rapid development of biotechnologies, there is still a large amount of data noise in the existing PPI network. Therefore, we propose an improved algorithm (RCRWR), which consists of network reconstruction based on local random walk dynamics and random walk with restart (see Algorithm 1 for the workflow of RCRWR). We try to use local random walks to extract the feature vectors of nodes (i.e., genes or proteins) and then use the feature vectors to calculate the similarity between nodes and reconstruct the PPI network to reduce the impact of data noise so as to improve the ability of disease-gene prediction based on the PPI network. Furthermore, we use KEGG pathways to enhance the ability to predict disease-related genes because the connections in the KEGG pathways tend to be stronger and more reliable than others.

**Table:** Algorithm 1 RCRWR Algorithm.

**Input:** PPIs, known disease genes, and number (*k*) of nearest neighbors.
**Output:** Probability scores.
1: Calculate behavior vectors (i.e., feature vectors) of all nodes by local random walk dynamics in the PPI network.
2: Calculate similarity scores between all nodes by the behavior vectors.
3: Generate a reconstructed PPI network by only retaining similarity scores between each node *i* (= 1~*n*) and its *k*-nearest neighbors.
4: Calculate probability scores of all nodes by applying network propagation based on random walk with restart to the reconstructed network, where known disease genes are used as seed nodes.

#### Network Reconstruction Based on Local Random Walks

##### Similarity Measure Based on Local Random Walk Dynamics

Generally, similar behavior patterns appear when the dynamic processes are triggered on similar nodes. Therefore, we applied the local random walk dynamics to infer the similarity measure between nodes (Lai et al., 2010; Xiang et al., 2016). The probability of a walker from one node to others in *k*-step random walk is determined by probability matrix *P*^*k*^ (*k* is random walk length, determining the range of the local structure that will be explored). Due to the small-world effect, good results can generally be generated by using a small *k*-value (*k* = 2, 3, …). The element *P*_*ij*_ of the transition matrix *P* is the ratio between the weight of link (*i, j*) and the weighted degree of vertex *i*, *P*_*ij*_ = *w*_*ij*_/∑_*j*_*w*_*ij*_, where *w*_*ij*_ is the weight of edge (*i, j*). The behaviors of the random walk dynamics from a node can be quantified by a *n*-dimensional vector *v*_*i*_ (*i* = 1~*n*; *n* is the number of nodes in a network), which is defined as the row of the matrix $\overset{k}{\underset{\tau =1}{\sum }}P^{\tau }$. Here, all random walks whose steps vary from 1 to *k* are taken into consideration to reinforce the contributions from the nodes near the target nodes. The similarity measure between nodes based on the local random walk dynamics can be calculated by,

(2) $$\begin{array}{r} S_{ij}=\frac{\left(v_{i},v_{j}\right)}{\sqrt{\left(v_{i},v_{i}\right)}\sqrt{\left(v_{j},v_{j}\right)}} \end{array}$$

where, if the behavior vectors *v*_*x*_ and *v*_*y*_ are highly consistent, then $s_{ij}^{}\to 1$; otherwise, $s_{ij}^{}\to 0$.

##### Network Reconstruction

We denote an undirected and weighted network by *G* = (*V, E, W*), where *V* is a set of proteins, *E* is a set of interactions, and *W* is a set of confidence scores of interactions in the original network. By using the above similarity measure based on local random walk dynamics (Equation 2), we calculate the similarity scores between all nodes in the original PPI network and obtain a similarity matrix *S*, where *S*_*ij*_ records the similarity score between nodes *i* and *j*. Then, we use the similarity scores to reconstruct the PPI network by retaining only the connections/similarity scores between each node *i* and its *k*-nearest neighbors (that is, its *k* neighbors with the highest similarity scores to the node *i*). The mathematical description of the reconstruction process is as follows.

**Definition 1**. For each node *i*, according to the similarity scores between the node and other nodes, all nodes are sorted in a descending order. By the descending order of all nodes, we define a ranking index vector, $R_{▪,i}^{}=\left\{R_{j,i}^{}|j=1,...,n\right\}$, to record ranking indices of all nodes about the node *i* (note that node *i* itself is given a largest ranking index), where *R*_*j, i*_ records the ranking index of node *j* in this case, and *n* is the number of nodes in the network.

**Definition 2**. By combining the ranking vectors about all nodes, we define a ranking matrix $R=\left(R_{▪,1}^{},R_{▪,2}^{},...,R_{▪,n}^{}\right)$, where *n* is the number of nodes in the network.

**Definition 3**. By using the ranking matrix and the similarity matrix *S*, we define a reconstructed and undirected network $Ĝ=\left(\hat{V},Ê,Ŵ\right)$, where $\hat{V}=V$, Ê and Ŵ denote the set of edges and the set of weights of edges in the reconstructed network, respectively:

$$\begin{array}{l} Ê=\left\{\left(j,i\right)|i=1\sim \;n,\;j=1\sim \;n,\;R_{j,i}^{}\leq k\;\right\},\\ Ŵ=\left\{S_{j,i}|i=1\sim n,j=1\sim n,R_{j,i}\leq k\right\}, \end{array}$$

where *S*_*j, i*_ = *S*_*i, j*_, and *k* denotes the number of the nearest neighbors (*k* = 50 for default).

In the reconstruction process for a given *k*-value, the newly added edges can be denoted by ${Ê}_{add}^{}=\left\{\left(j,i\right)|i=1\sim \;n,\;j=1\sim \;n,\;R_{j,i}^{}\leq k\;and\;\left(j,i\right)\notin E\right\}$; the removed edges can be denoted by ${Ê}_{remove}^{}=E\backslash Ê$; the retained edges can be denoted by ${Ê}_{retain}^{}=E⋂Ê$; and the weights of the retained edges are substituted by the similarity scores obtained by the similarity measure based on local random walk dynamics.

By using the reconstruction process, we can generate a reconstructed and undirected network. The reconstructed network may enhance our ability for disease-gene prediction because it can improve the original PPI network. To show the effect of the reconstruction process on the PPI network, we have generated a set of reconstructed PPI networks by using a series of *k*-values, and then we calculate the mean score (in the String database) of retained edges ${Ê}_{retain}^{}$ and removed edges ${Ê}_{remove}^{}$ for each *k* value. The results show that the mean score (in the String database) of the retained edges tends to be larger than that of the removed edges (see Supplementary Figure 1). This is consistent with our expectation: By using the reconstruction process, PPIs with high reliability in the String database tend to be retained, and PPIs with low reliability in the String database tend to be removed, and the reconstruction process also supplements some edges with high similarity scores that do not exist in the original PPI network. Moreover, we have provided an example figure to compare the original network with the reconstructed one, which shows the effect of network reconstruction on the original network, so the reader can more clearly see what is being done (see Supplementary Figure 2).

As a whole, this reconstruction process may reduce data noise to a certain extent to optimize the PPI network so as to improve the network data environment for disease-gene prediction. In the following step, we apply network propagation to the reconstructed network to predict disease-related genes more effectively.

#### Network Propagation Based on Random Walk With Restart

The random walk with restart can been seen as performing multiple random walks over the PPI network, each starting from a seed node associated to a known disease gene, iteratively moving from one node to a random neighbor, and the stationary distribution can be considered as a measure of the proximity between the seed(s) and all the other nodes in the network. More formally, the random walk with restart is defined as

(3) $$\begin{array}{r} p_{t+1}^{T}=\left(1-r\right)Mp_{t}^{T}+rp_{0}^{T} \end{array}$$

Here, *p*_0_ is the initial probability distribution. *M* is the column-normalized adjacency matrix of the graph. *r*∈(0, 1) is the restart probability, and it is set to be 0.7 as suggested by previous studies (Zhao et al., 2015). *p*_*t*_ is the probability vector of the random walker reaching all nodes at the end of the *t*th step. After several iterations, the difference between the vectors *p*_*t*+1_ and *p*_*t*_ becomes negligible, the stationary probability distribution is reached, and the element in the vector represents a proximity measure between every graph node and the seed(s). In this work, iterations are repeated until the difference between *p*_*t*_ and *p*_*t*+1_ falls below 10^-6^ as used by previous studies (Zhao et al., 2015).

Note that for cross-validation, the known disease-related genes in the training set are used as seed nodes to conduct the random walk with restart, and all known disease-related genes are used as seed nodes when predicting novel candidate genes.

#### Prediction Based on PPI Network

We first prepare the PPI network. The PPI network from the String database retains edges with confidence scores >400, and we normalize the confidence scores to be between zero and one by dividing a value of 1,000. The PPI network is used as the original PPI network. We use a weighted graph *G* = (*V, E, W*) to denote the PPI network comprising a set of proteins *V*, a set of interactions *E*, and a set of confidence scores *W*. Then, we map known breast cancer–related genes into the PPI network and conduct the random walk with restart to predict disease-related genes. Finally, the probabilities of nodes are used to rank candidate genes.

#### Prediction Based on PPI Network and KEGG Pathway

Similarly, we prepare the related data sets, including the PPI network, breast cancer–related genes, and KEGG pathway. The PPI network still retains edges with confidence >400. We map known breast cancer–related genes to the PPI network. Then, KEGG pathways are mapped into the PPI network and intersect with the above network. Finally, we perform the random walk with restart to predict breast cancer–related genes.

#### Performance Evaluation

To evaluate the prediction performance of the algorithm, we apply traditional 3-fold cross-validation in the benchmark. Each time, the known disease genes are randomly split into three parts. Each part is, in turn, used as test set and the rest as a training set. Then, we use the genes in the training set as seeds to perform the random walk with restart to predict disease-related genes. Note that, in the process of predicting disease genes, only genes in the training set are used as seed genes. For the cross-validation, the training set made up of two thirds of all disease genes randomly selected. For the prediction of novel genes, all known disease genes are used as the training set.

For a disease *d* in disease set *D*, *T*_*d*_ denotes the set of genes in test set. The disease-gene prediction algorithm provides a ranking list of candidate genes for disease *d*. We denote by *R*_*d*_(*k*) the set of top *k* candidate genes in the ranking list. Then recall in the top *k* ranking list is defined as

(4) $$\begin{array}{r} Recall\;\left(k\right)=\frac{\left|T_{d}\cap R_{d}\left(k\right)\right|}{\left|T_{d}\right|} \end{array}$$

This metric is used to evaluate the performance of prediction algorithms.

## Results

**Figure 1:**
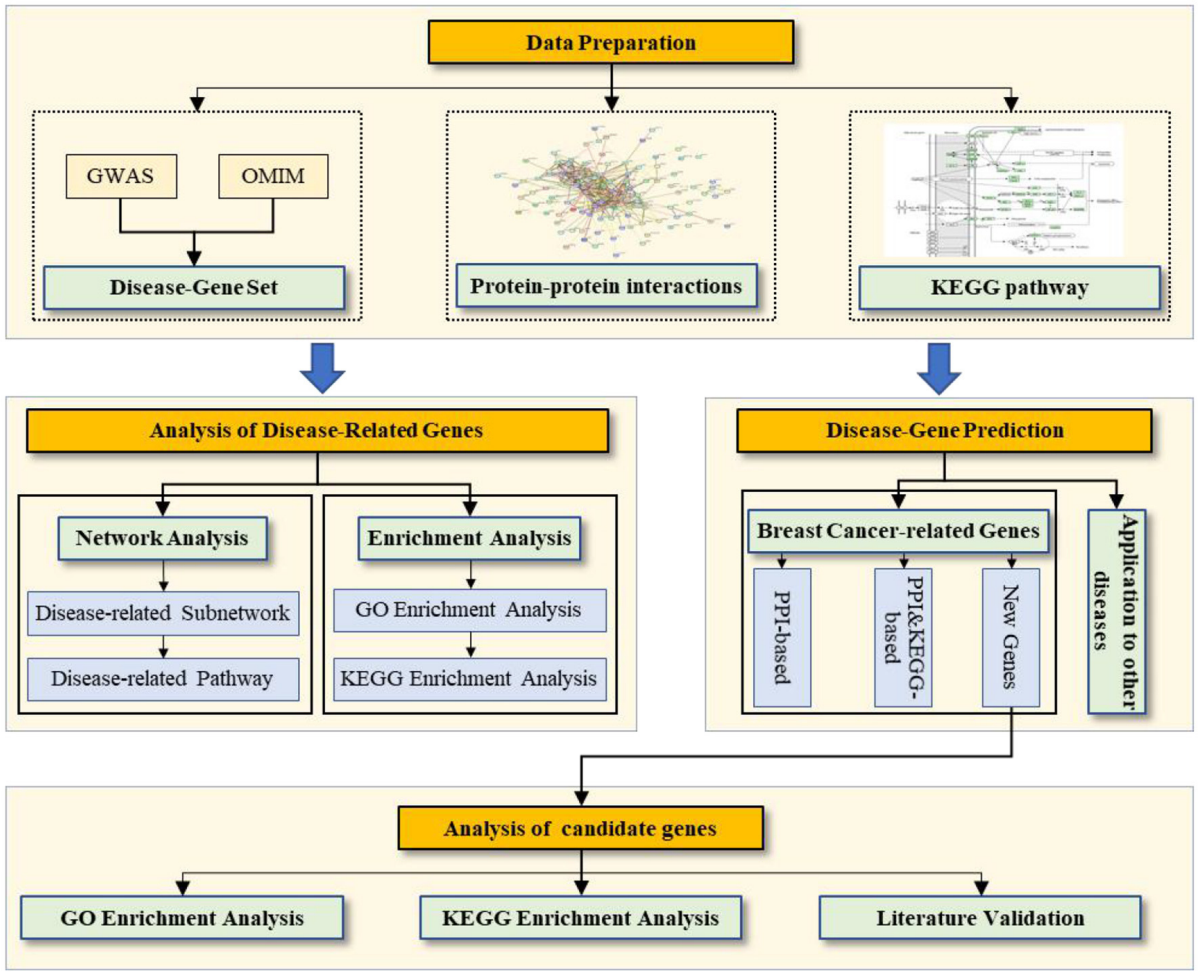
Workflow of the work.

Here, we first conduct two types of analysis for breast cancer–related genes: (1) network analysis of the breast cancer–related subnetwork and KEGG pathways and (2) enrichment analysis of GO and the pathway of breast cancer–related genes. Then, we predict breast cancer–related genes on the (reconstructed) PPI network with and without the KEGG pathways and analyze the prediction performance, including (1) quantitative evaluation on the known breast cancer–related gene set, (2) enrichment analysis of GO and the pathway of candidate genes, and (3) a literature validation of candidate genes. Figure 1 shows the workflow.

### Analysis of Breast Cancer-Related Genes

#### Network Analysis

##### Subnetwork of Breast Cancer-Related Genes

**Figure 2:**
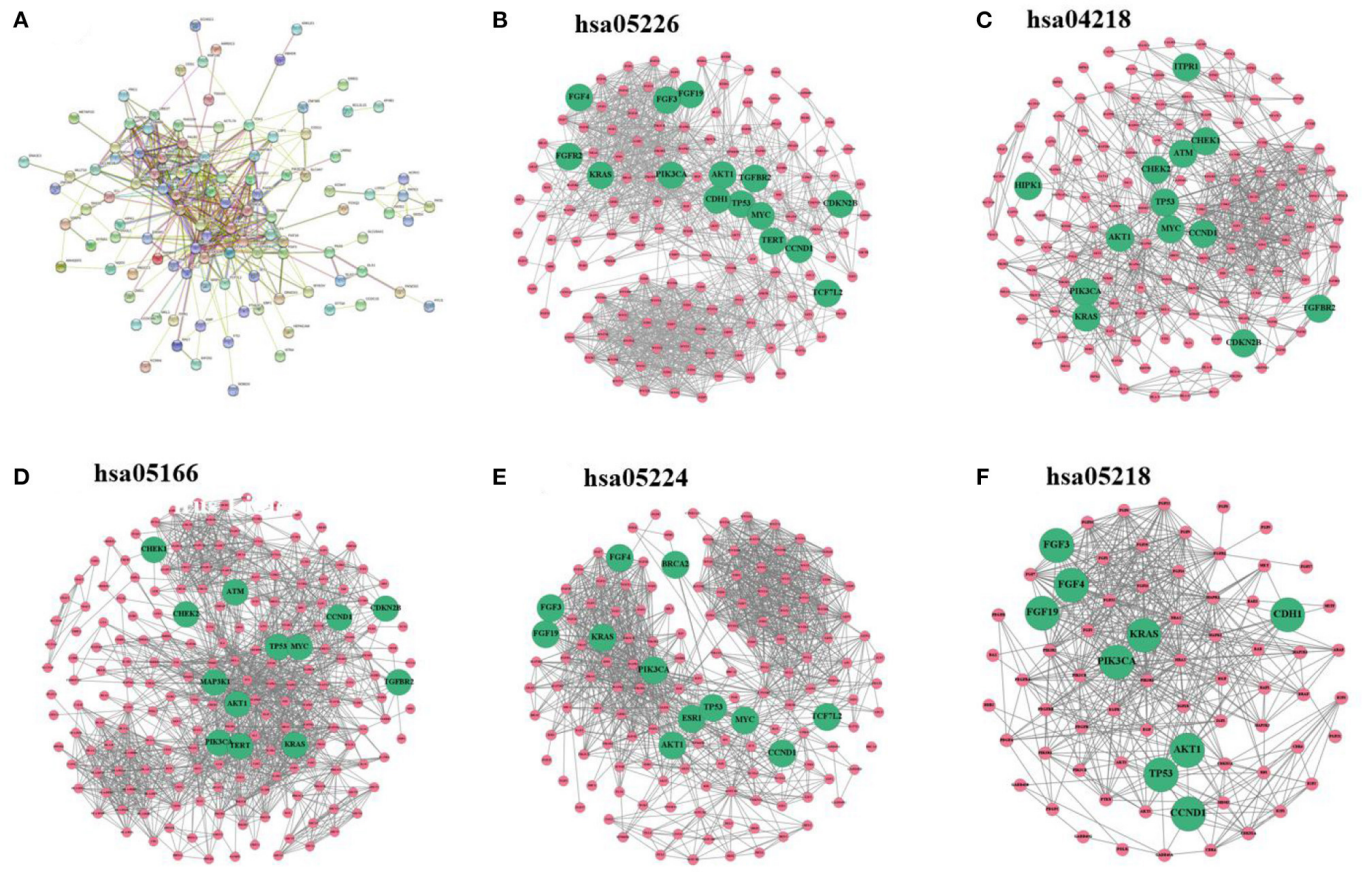
**(A)** Subnetwork of breast cancer–related genes extracted from the PPI network. **(B–F)** Subnetworks extracted from the PPI network by using the sets of genes of five KEGG pathways, respectively: gastric cancer, cellular senescence, human T cell leukemia virus 1 infection, breast cancer, and melanoma. Note that green nodes with larger size denote known breast cancer–related genes.

Breast cancer–related genes were obtained from Yang et al. (2016). After mapping breast cancer–related genes into the PPI network, there are only 127 breast cancer–related genes. We first analyze the distribution of breast cancer–related genes in the PPI network as well as KEGG pathways (Figure 2). Supplementary Figures 3–7 provide larger plots so that gene names can be identified more easily. Figure 2A displays the subnetwork of breast cancer–related genes. The subnetwork is extracted from the PPI network by only retaining breast cancer–related genes. We quantitatively analyze the breast cancer–related subnetwork by calculating six statistical measures of networks (see Table 1). We find that the breast cancer–related subnetwork has a higher value of the clustering coefficient (CC) and higher link density compared with random sampling on the whole network, showing significantly more interactions than expected. These results quantitatively suggest that the breast cancer–related genes/proteins tend to interact with each other, forming disease module with higher link density than expected.

**Table 1:** Statistics of disease-gene subnetworks related to breast cancer as well as other diseases.

**Disease**	**#Genes**	**#Interactions**	**Degree**	**CC**	**Link density**	***p-*value**
Breast cancer	130	477 (232 ± 24)	7.3	0.55 (0.42 ± 0.04)	5.7% (2.8% ± 0.3%)	<1.0e-16
Rheumatoid arthritis	115	607 (87 ± 15)	10.6	0.45 (0.36 ± 0.04)	9.3% (1.3% ± 0.2%)	<1.0e-16
Cholesterol	221	1,152 (245 ± 27)	10.4	0.47 (0.37 ± 0.03)	4.7% (1.0% ± 0.1%)	<1.0e-16
Obesity	102	764 (65 ± 14)	15.0	0.62 (0.35 ± 0.05)	14.8% (1.3% ± 0.3%)	<1.0e-16
Hypertension	104	234 (64 ± 9)	4.5	0.44 (0.35 ± 0.05)	4.4% (1.2% ± 0.2%)	<1.0e-16
Metabolic traits	135	439 (70 ± 10)	6.5	0.38 (0.34 ± 0.04)	4.9% (0.8% ± 0.1%)	<1.0e-16
Crohn's disease	194	847 (198 ± 27)	8.7	0.50 (0.38 ± 0.04)	4.5% (1.1% ± 0.1%)	<1.0e-16
Inflammatory bowel disease	220	1,653 (251 ± 32)	15.0	0.52 (0.38 ± 0.03)	6.9% (1.1% ± 0.1%)	<1.0e-16
Metabolite levels	95	366 (44 ± 10)	7.7	0.50 (0.34 ± 0.05)	8.2% (1.0% ± 0.2%)	<1.0e-16
Prostate cancer	238	589 (300 ± 24)	5.0	0.44 (0.39 ± 0.03)	2.1% (1.1% ± 0.1%)	<1.0e-16

*Disease-gene subnetworks are extracted from the PPI network by retaining genes related to specific disease, e.g., breast cancer. #Genes and #Interactions denote the number of genes and edges in the subnetworks, respectively. Degree and CC denote the average degrees of all nodes and CCs in the subnetwork, respectively. Link density is defined as ratio of the number of existing interactions to its maximum of possible edges in the subnetwork. p-value evaluates the significance of interaction enrichment in the subnetwork. “(x ± y)” denotes the mean and standard deviation of statistics in random sampling*.

As we know, in PPI networks, proteins with similar functions tend to connect or interact with each other. The occurrence and development of disease is usually due to the abnormal function of related genes or proteins, which leads to the change of related signal pathways. These proteins usually have functional similarity or correlation. Therefore, genes of the same disease or similar diseases tend to connect with each other in the PPI network to form disease modules.

We calculate the six statistical measures for subnetworks of other diseases, such as rheumatoid arthritis, cholesterol, and obesity (see Table 1). Similar conclusions can be obtained for other diseases. Clearly, these diseases also have similar modular property. This again confirms the modular property of disease-related genes (Ghiassian et al., 2015; Xiang et al., 2016; Chen et al., 2018; Hu et al., 2018, 2020; Choobdar et al., 2019; Dwivedi et al., 2020). This is why guilt by association can become a useful strategy in disease-gene prediction based on PPI networks.

##### Subnetworks of KEGG Pathways Related to Breast Cancer

Moreover, we study subnetworks of KEGG pathways related to breast cancer. We analyze the distribution of breast cancer–related genes in KEGG pathways (also, see Supplementary Figures 1–5).

We extract the subnetworks of the KEGG pathways from the PPI network by using the sets of genes of the KEGG pathways and calculate the statistical measures of networks for these subnetworks. Table 2 lists five KEGG pathways significantly related to breast cancer along with the statistical measures of the subnetworks. The results show that these subnetworks have similarly higher values of CC and higher link density than the whole network, and it has significantly more interactions than expected (*p* <1.0e-16). This means the genes/proteins in these KEGG pathways also tend to interact with each other, forming modules with higher link density than expected.

**Table 2:** Statistics of KEGG pathways related to breast cancer.

**Pathway ID**	**Pathway Name**	**#Matched Genes**	**#Genes**	**#Interactions**	**Degree**	**CC**	**Link density**	***p*-value**
hsa04218	Cellular senescence	13	156	2,377 (1,136 ± 69)	30.5	0.65(0.52 ± 0.02)	19.7% (9.6% ± 0.6%)	<1.0e-16
hsa05224	Breast cancer	12	147	3,169 (1,059 ± 79)	43.1	0.72(0.57 ± 0.03)	29.5% (9.9% ± 0.7%)	<1.0e-16
hsa05226	Gastric cancer	15	149	3,042 (953 ± 74)	40.8	0.71(0.55 ± 0.03)	27.6% (9.0% ± 0.7%)	<1.0e-18
hsa05166	Human T-cell leukemia virus 1 infection	13	217	3,872 (1,516 ± 96)	35.7	0.63(0.49 ± 0.02)	16.5% (6.6% ± 0.4%)	<1.0e-17
hsa05218	Melanoma	9	72	1,112 (385 ± 39)	30.9	0.77(0.61 ± 0.04)	43.5% (15.1% ± 1.5%)	<1.0e-16

*The KEGG pathways used in analysis are selected based on the number of matched genes between the pathways and known disease gene set.*

*#Matched Genes denotes the number of common genes between gene set of pathway and breast cancer–related gene set; #Genes in Pathway denotes the number of genes in pathway; #Edges denotes the number of interaction in the PPI subnetwork consisting of genes in pathway; Degree and CC denote the average degrees of all nodes and CCs in the subnetwork, respectively. Link density is defined as ratio of the number of existing interactions to its maximum of possible edges in the subnetwork. p-value evaluates the significance of interaction enrichment in the subnetwork. “(x ± y)” denotes the mean and standard deviation of statistics in random sampling*.

The values of CC and link density for most KEGG pathways are higher than those of the above subnetwork of breast cancer–related genes (see Tables 1, 2). This means the genes in the KEGG pathways are more modular than breast cancer–related genes. The reason may be that genes in these KEGG pathways are more closely related than other genes in functions. Moreover, we can find that there exist submodule structures in the subnetworks of the KEGG pathways (see Figures 2B–F). This means that there exist functional subunits in the KEGG pathways.

We label known breast cancer–related genes in the subnetworks of the KEGG pathways. Other unlabeled genes in the KEGG pathways are also likely to be related to breast cancer because they are likely to jointly affect breast cancer–related functions. One can see that some subunits have more breast cancer–related genes. This means that the known breast cancer–related genes may be non-randomly distributed in the subnetworks of KEGG pathways, and some subunits in the KEGG pathways may be more related to breast cancer.

Overall, the physical and functional connections between genes in the KEGG pathways are stronger and more reliable than others. Therefore, we make use of information of KEGG pathways in disease-gene prediction.

#### Enrichment Analysis

**Figure 3:**
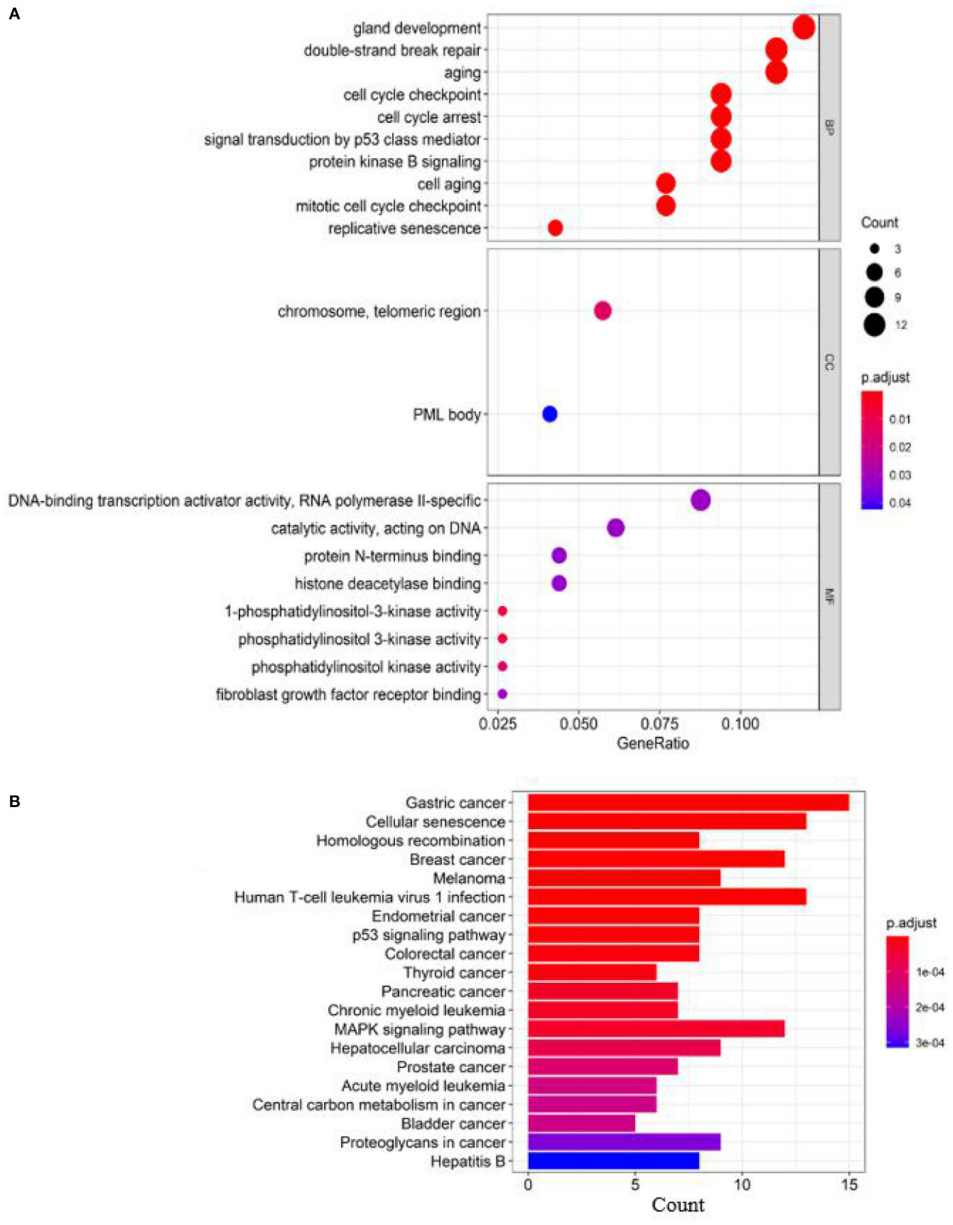
Enrichment analysis of known breast cancer–related genes: **(A)** GO enrichment analysis and **(B)** pathway enrichment analysis.

To analyze the relatedness of disease-gene sets to functional units, we perform GO enrichment analysis and KEGG pathway enrichment analysis. Figure 3 shows the results of GO enrichment analysis and KEGG pathway enrichment analysis (obtained by clusterProfiler; Yu et al., 2012).

According to the GO terms in Figure 3, breast cancer–related genes are enriched in the following GO terms, e.g., “double-strand break repair,” “replicative senescence,” “cell aging,” “aging,” “cell cycle checkpoint,” “cell cycle arrest,” “gland development,” “signal transduction by p53 class mediator,” “mitotic cell cycle checkpoint,” and “protein kinase B signaling.”

According to the KEGG pathways in Figure 3, breast cancer–related genes are enriched in cancer-related KEGG pathways, e.g., gastric cancer, endometrial cancer, colorectal cancer, thyroid cancer, pancreatic cancer, prostate cancer, central carbon metabolism in cancer, proteoglycans in cancer, bladder cancer.

BRCA gene mutations, which are commonly present in breast cancer, are associated with significantly increased susceptibility to tumors, including prostate, pancreatic, gallbladder/cholangioma, and stomach cancer as well as malignant melanoma. These tumors share a common pathogenic gene network in which the BRCA gene plays an important role as it is a member of the mismatch repair gene family. The prediction of breast cancer–related genes can discover the interaction between tumors and enrich the relationship network, which is of great significance for finding therapeutic targets for tumors.

### Prediction of Breast Cancer Genes Based on PPI Network

**Figure 4:**
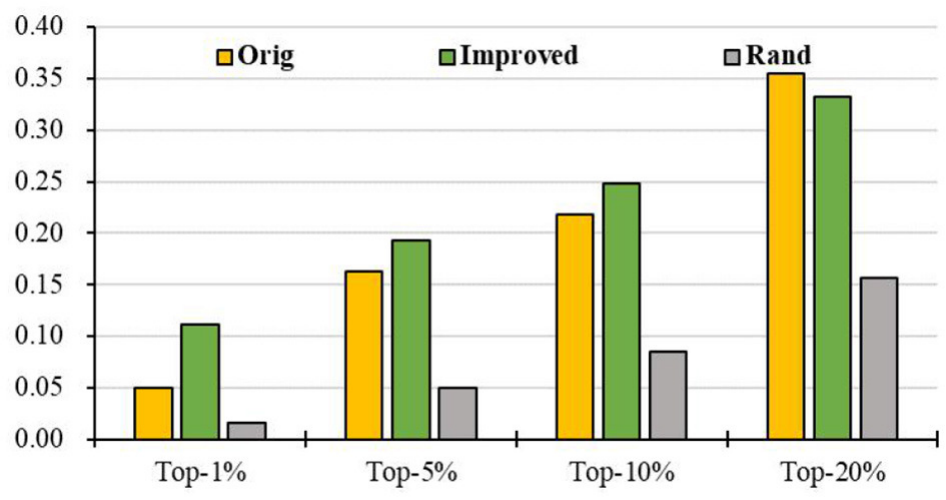
Top-*k* Recall (*k* = 1, 5, 10, 20%) of the original and improved algorithms in the PPI network.

To evaluate the prediction performance of our algorithm, we first apply RCRWR to the PPI network. The results show that RCRWR significantly outperforms the original RWR algorithm (Wu et al., 2008) on the PPI network for the top 1, 5, and 10% lists of candidate genes (see Figure 4). This means that the network reconstruction indeed can improve the PPI network so as to enhance the ability to predict breast cancer–related genes. Moreover, it is clear that RCRWR and RWR are significantly better than that in the random case.

### Prediction of Breast Cancer Genes Based on PPI Network and KEGG Pathway

**Figure 5:**
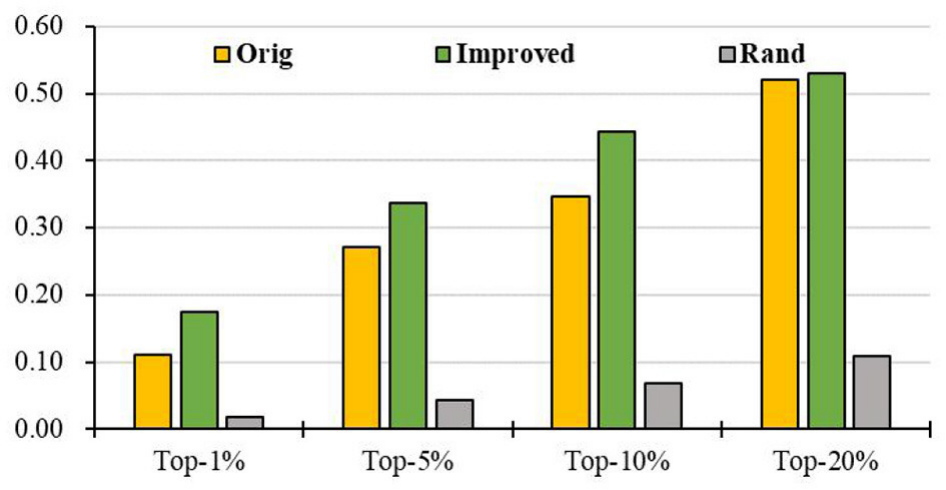
Top-*k* Recall (*k* = 1, 5, 10, 20%) of the original and improved algorithms in the PPI network with KEGG pathway (PPI_ KEGG).

Further, we intersect genes in the KEGG pathways with genes in the PPI to obtain a more reliable PPI network and then apply RCRWR to the PPI network. The results show that RCRWR is significantly better than the RWR algorithm on the PPI network for top 1, 5, 10, and 20% lists of candidate genes (see Figure 5). This again proves that the network reconstruction can indeed enhance the ability to infer breast cancer–related genes on the PPI network. Moreover, it is clear that the results of RCRWR and RWR are also significantly better than in the random case.

**Figure 6:**
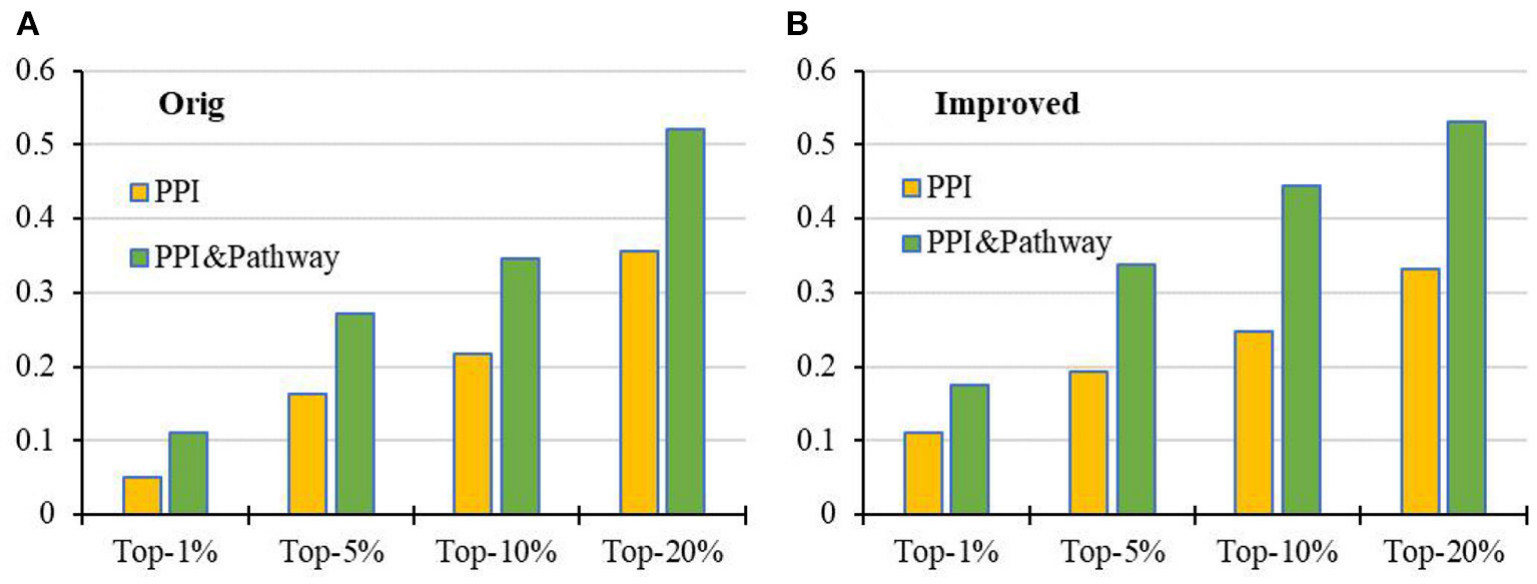
Comparison of top-*k* Recall (*k* = 1, 5, 10, 20%) in the PPI network with and without KEGG pathway by the **(A)** original algorithm and **(B)** improved algorithm.

Compared with the results on the PPI network with and without KEGG pathway data (see Figure 6), it is very clear that the prediction performance of both RWR and RCRWR can be enhanced due to the addition of information of the KEGG pathway. The information of the KEGG pathway is very helpful for the prediction of disease-related genes.

### Analysis of Candidate Genes of Breast Cancer

Here, we use all known breast cancer–related genes as training set to predict candidate genes. We map breast cancer–related genes into the PPI network and map the KEGG pathway onto the PPI network because the KEGG pathway is helpful for disease-gene prediction. We perform our improved algorithm RCRWR in the network to score all candidate genes. Then, we generate a ranking list of candidate genes for breast cancer. The higher the ranking of genes, the more likely they are to be associated with breast cancer.

We list the top 10 predicted genes in Table 3, which are considered to be most closely associated with breast cancer according to the scores from prediction algorithm. To check the effectiveness of prediction for the candidate genes, we search the literature and try to find the connections between these genes and breast cancer.

**Table 3:** Predicted top 10 candidate genes for breast cancer using PPI and KEGG pathway.

**Gene**	**References**
*CDK4*	Ullah Shah et al., 2015
*RAD51*	Gao et al., 2011; Wong et al., 2011; Wu et al., 2015; Liang et al., 2016; Bhattacharya et al., 2017
*ATR*	Di Benedetto et al., 2017
*TOP3A*	Broberg et al., 2009
*BLM*	Ding et al., 2009
*XRCC6*	Willems et al., 2009; He et al., 2012
*RAD52*	Huang et al., 2016
*EXO1*	Wang et al., 2009
*MRE11A*	Podralska et al., 2018
*RAD54B*	Zhang et al., 2019

DNA damage repair is an important cellular defense mechanism, and its dysfunction has been linked to a variety of diseases, including breast cancer. Most of the top 10 candidate genes for breast cancer are related to the DNA damage repair function. RAD51 is a eukaryotic protein that plays a role in DNA repair, neuronal development in the motor system, and innate immune response (Liang et al., 2016). At present, studies on the RAD51 gene mainly focus on the interaction between tumor suppressors, the cell cycle, and apoptotic regulators to promote the transformation of normal breast epithelial cells into tumor molecules (Bhattacharya et al., 2017). Genetic association studies confirm that the RAD51 polymorphisms contribute to the susceptibility of breast cancer in multiple populations (Gao et al., 2011; Wong et al., 2011; Wu et al., 2015). RAD52 and RAD54B are key homologous recombination repair (HRR) proteins, which is closely related to the annealing of homologous complementary sequences. RAD52 is shown to be associated with breast cancer susceptibility genes BRCA1 and BRCA2. When RAD52 is knocked out in BRCA1- or BRCA2-deficient tumor cells, HRR frequency is significantly reduced (Huang et al., 2016). For RAD54B, Zhang et al. show that RAD54B protein expression in breast cancer tissues was higher than that in adjacent normal tissues through bioinformatics analysis of multiple relevant databases and experiments related to immunohistochemistry and breast cancer cell lines (Zhang et al., 2019). In addition, the X-ray repair cross-complementing 6 (XRCC6) protein was also a key molecule on the non-homologous end-joining (NHEJ) repair pathway (Bau et al., 2011). Studies show that the XRCC6 polymorphism is correlated with the occurrence and development of breast cancer (Willems et al., 2009; He et al., 2012). Ataxia-telangiectasia mutated and Rad3-related protein (ATR) is an important regulator of the response mechanism of DNA damage repair. The ATR molecular pathway regulates cell DNA damage repair through a variety of cytokines, thus leading to the development of normal cells into tumor cells. High ATR expression was found to be associated with late breast cancer stage and poor prognosis (Di Benedetto et al., 2017). Furthermore, Exonuclease 1 (EXO1), a kind of multifunctional enzyme, is mainly used in clearing double-stranded DNA or RNA molecules that exist in the single sequence. Wang et al. report that the A allele EXO1 K589E conferred a significantly increased risk of breast cancer (Wang et al., 2009). Apart from the above genes, the CDK4 (Ullah Shah et al., 2015), MRE11A (Podralska et al., 2018), BLM (Ding et al., 2009), and TOP3A (Broberg et al., 2009) are shown to be associated with the pathogenesis of breast cancer. These results show that our predictions are in concert with existing reports, and the algorithm is valuable for predicting the new disease-gene associations.

**Figure 7:**
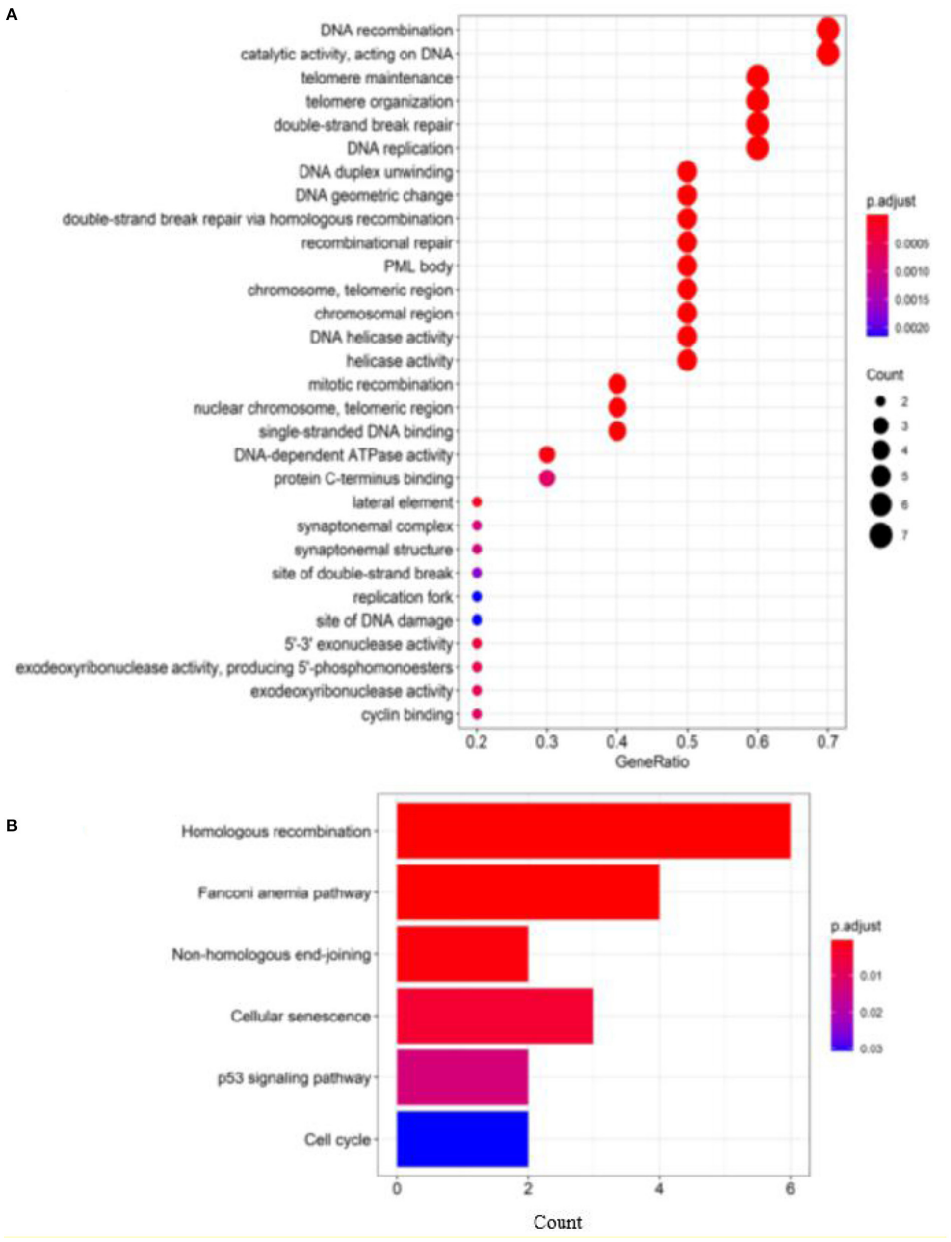
Enrichment analysis of top 10 candidate genes for breast cancer: **(A)** GO enrichment analysis and **(B)** pathway enrichment analysis.

To further evaluate our predictions, we perform GO and KEGG pathway enrichment analysis on the top 10 ranked genes. The results of GO enrichment analysis show that the genes are mostly enriched in DNA recombination in its biological process, PML body in its cellular component and catalytic activity, acting on DNA in its molecular function (Figure 7A). GO analysis shows that these genes are involved in DNA damage repair and cell growth and transformation, which are important in the pathogenesis of cancers. According to the KEGG pathways listed in Figure 7B, the top 10 candidate genes are enriched in cells divide and grow pathways including homologous recombination, NHEJ, and cell cycle pathways, which are shown to play important roles in the division and growth of cancer cells.

### Application to Other Diseases

**Figure 8:**
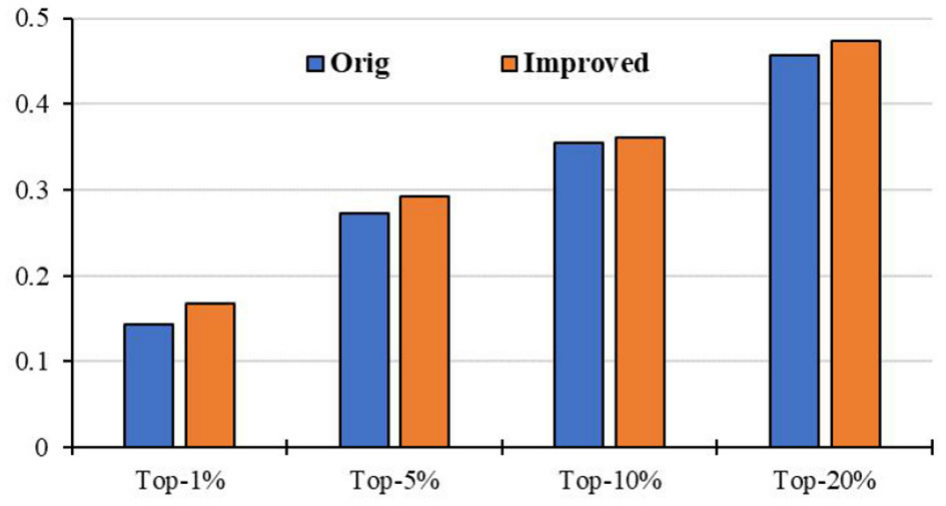
Top-*k* Recall performance for all diseases in the PPI network.

**Figure 9:**
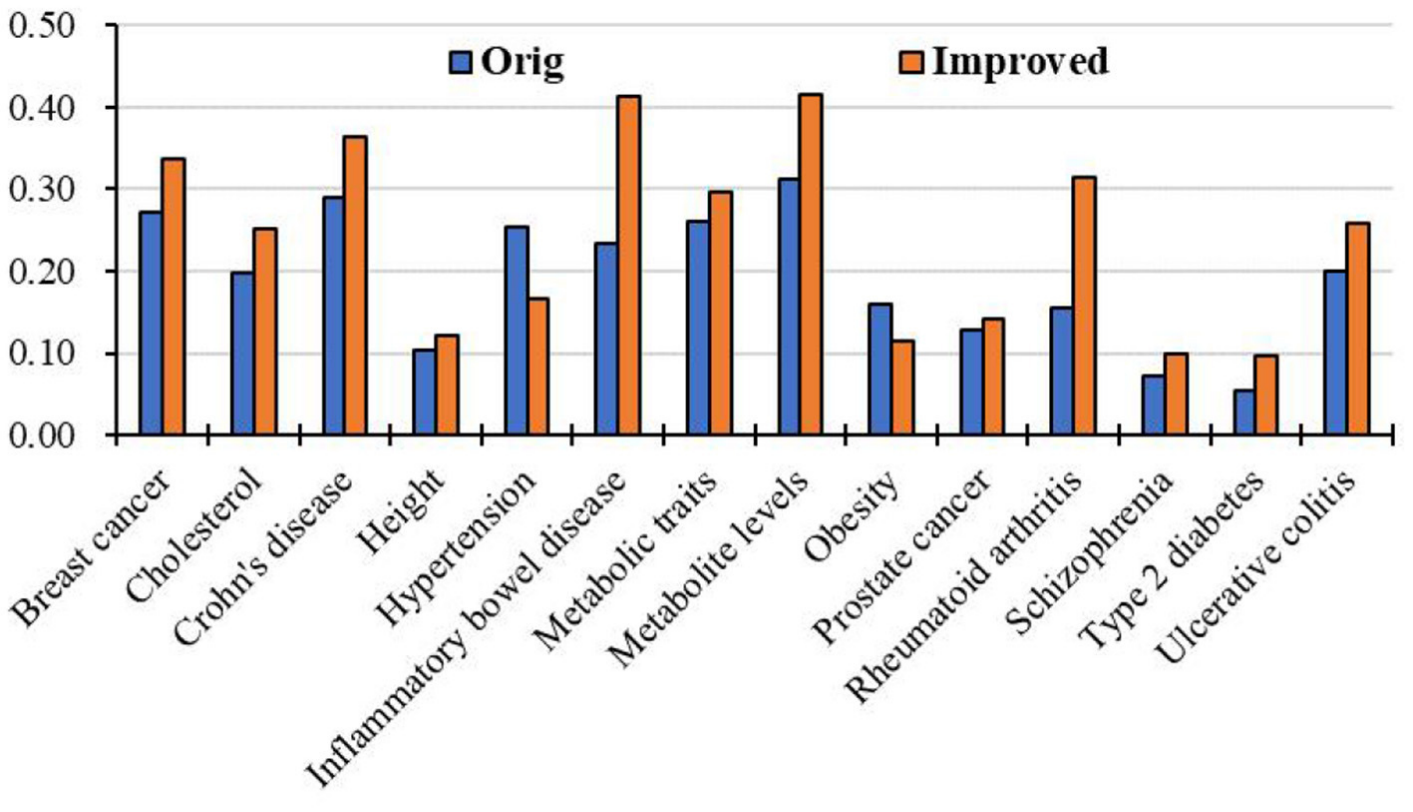
Top 5% Recall for different diseases in the PPI network with KEGG pathway.

Moreover, we apply the above RCRWR algorithm to other diseases, such as inflammatory bowel disease, metabolite levels, and cholesterol. To display the prediction performance in the diseases, we still apply 3-fold cross-validation to the diseases. Figure 8 shows average top-*k* Recall prediction performance for all diseases in the data set. The results show that RCRWR outperforms the original algorithm on the whole. As examples, Figure 9 shows the top 1% Recall prediction performance for some diseases. The results show that RCRWR can improve the ability of predicting disease-related genes for most diseases such as inflammatory bowel disease and rheumatoid arthritis.

## Conclusion

In this study, we have conducted analysis and prediction of breast cancer–related genes based on the PPI network and KEGG pathway. First, we analyzed the distribution of breast cancer–related genes from the aspects of network and enrichment analysis. The results show that the subnetwork of breast cancer–related genes has larger link density than that of the whole network. This means that the breast cancer–related genes tend to cluster together in the network, forming a disease module related to breast cancer. This is the case for other diseases. We also analyzed the structures of the KEGG pathways significantly related to breast cancer and visually display the distribution of breast cancer–related genes in KEGG pathways, which may help to understand how breast cancer–related genes affect related biological processes and functions in breast cancer.

Further, we propose the improved algorithm RCRWR to predict genes related to breast cancer as well as other diseases in the PPI network with and without the KEGG pathway. The results show that RCRWR can effectively improve the ability of predicting genes related to breast cancer and other diseases in the PPI network, and the KEGG pathway is very useful in enhancing disease-gene prediction. We used known breast cancer–related genes as a training set to predict candidate genes. For the top 10 candidate genes, we conducted enrichment analysis of the GO and KEGG pathways as well as literature validation and confirmed the connections between these candidate genes and breast cancer. This means that the list of candidate genes is closely related to breast cancer. We believe that these results may provide useful insights into the study of breast cancer–related genes and the understanding of its molecular mechanism.

## Data Availability Statement

The original contributions presented in the study are included in the article/Supplementary Material, further inquiries can be directed to the corresponding author/s.

## Author Contributions

JY, B-SH, and JL conceived, designed, and managed the study. YZ and JX performed the experiments and drafted the manuscript. LT, JL, QL, and GT reviewed the manuscript. All authors approved the final manuscript.

## Conflict of Interest

JY, GT, and QL were employed by the company Geneis Beijing Co., Ltd. The remaining authors declare that the research was conducted in the absence of any commercial or financial relationships that could be construed as a potential conflict of interest.

## Publisher's Note

All claims expressed in this article are solely those of the authors and do not necessarily represent those of their affiliated organizations, or those of the publisher, the editors and the reviewers. Any product that may be evaluated in this article, or claim that may be made by its manufacturer, is not guaranteed or endorsed by the publisher.
