# Supplementary material for: Identifying Breast Cancer-Related Genes Based on a Novel Computational Framework Involving KEGG Pathways and PPI Network Modularity
Source: Front Genet. 2021 Aug 16;12:596794. doi: 10.3389/fgene.2021.596794 (PMC8415302; doi:10.3389/fgene.2021.596794)
Supplement: Supplementary file 1 [file Datas_Sheet_1.docx]

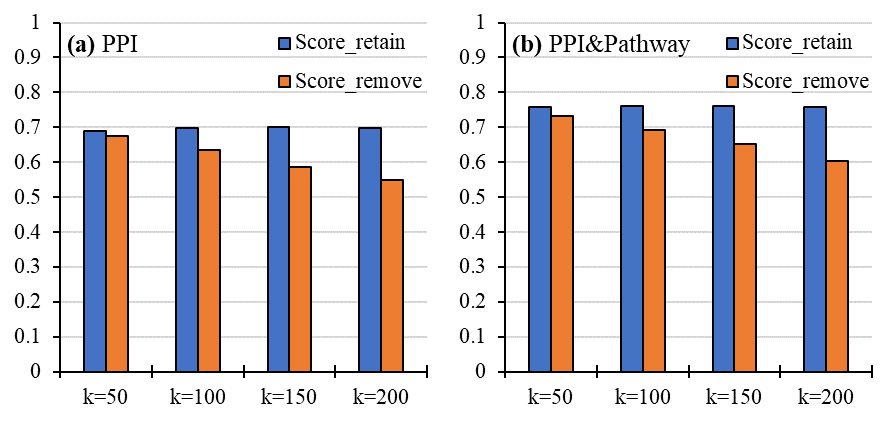


**Figure S1**. After network reconstruction by using different k-values, comparison of mean score (in String database) of retained edges with that of removed edges in (a) the PPI network and (b) the PPI network with Pathways.


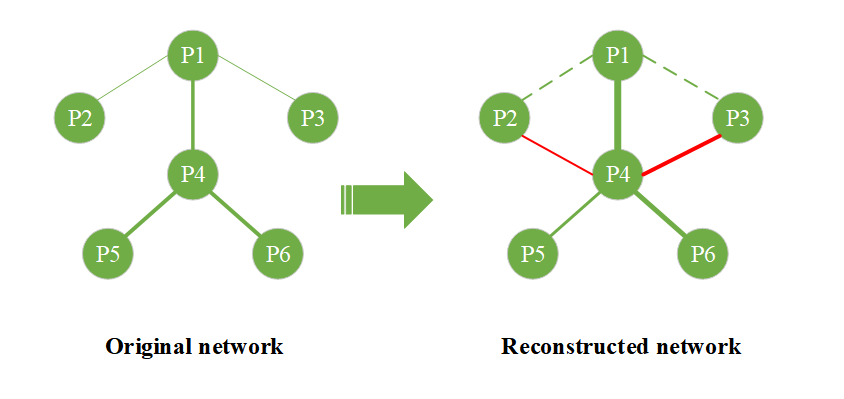


**Figure S2**.Example networks show the effect of network reconstruction on the original network, so that the reader can more clearly see what is being done in network reconstruction. P1-P6 denote nodes (proteins or genes) in the network. The interactions are marked with blue lines and thicker lines denote edges with higher confidence or higher similarity scores.

**[Appendix ] Subnetworks extracted from the PPI network by using the sets of genes of five KEGG pathways**

**hsa05226 Gastric cancer**


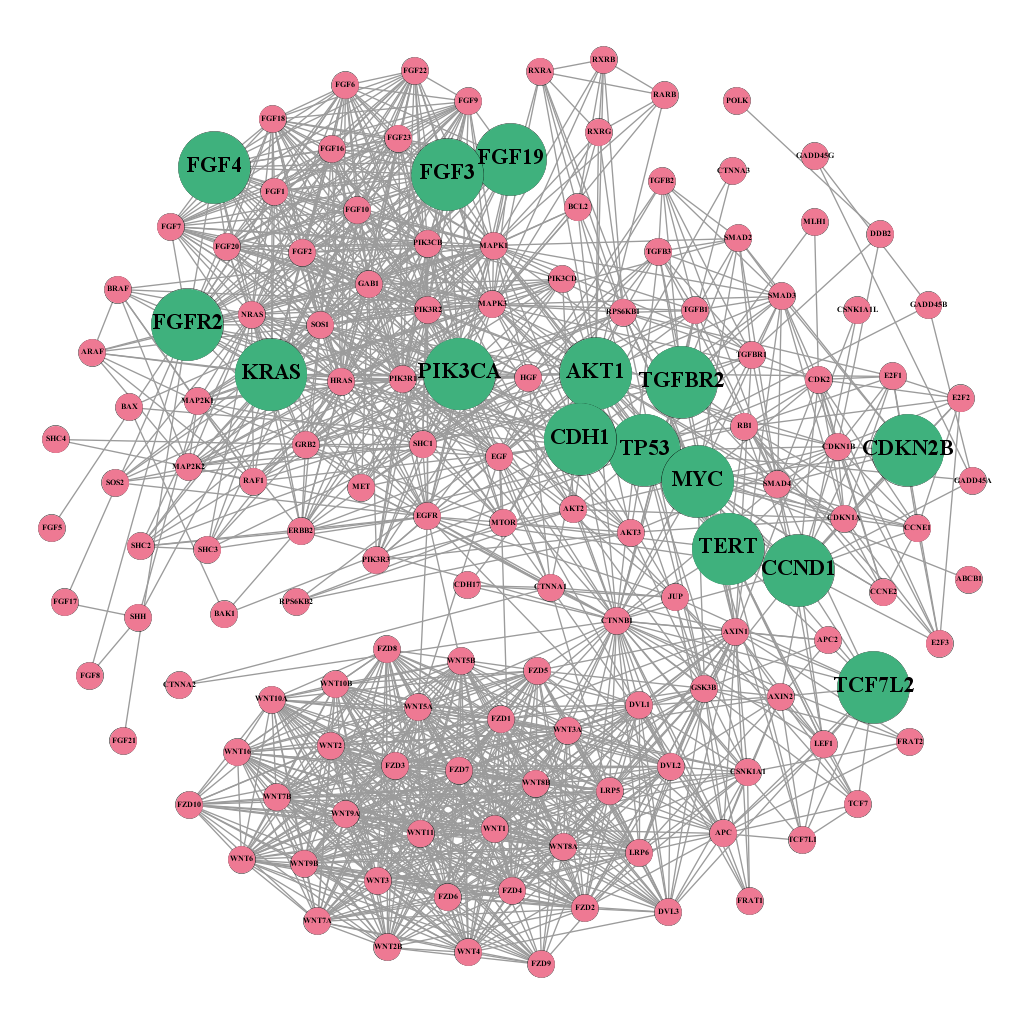


**Figure S3**. Subnetwork extracted from the PPI network by using the sets of genes of hsa05226: Gastric cancer.

**hsa04218 Cellular senescence**


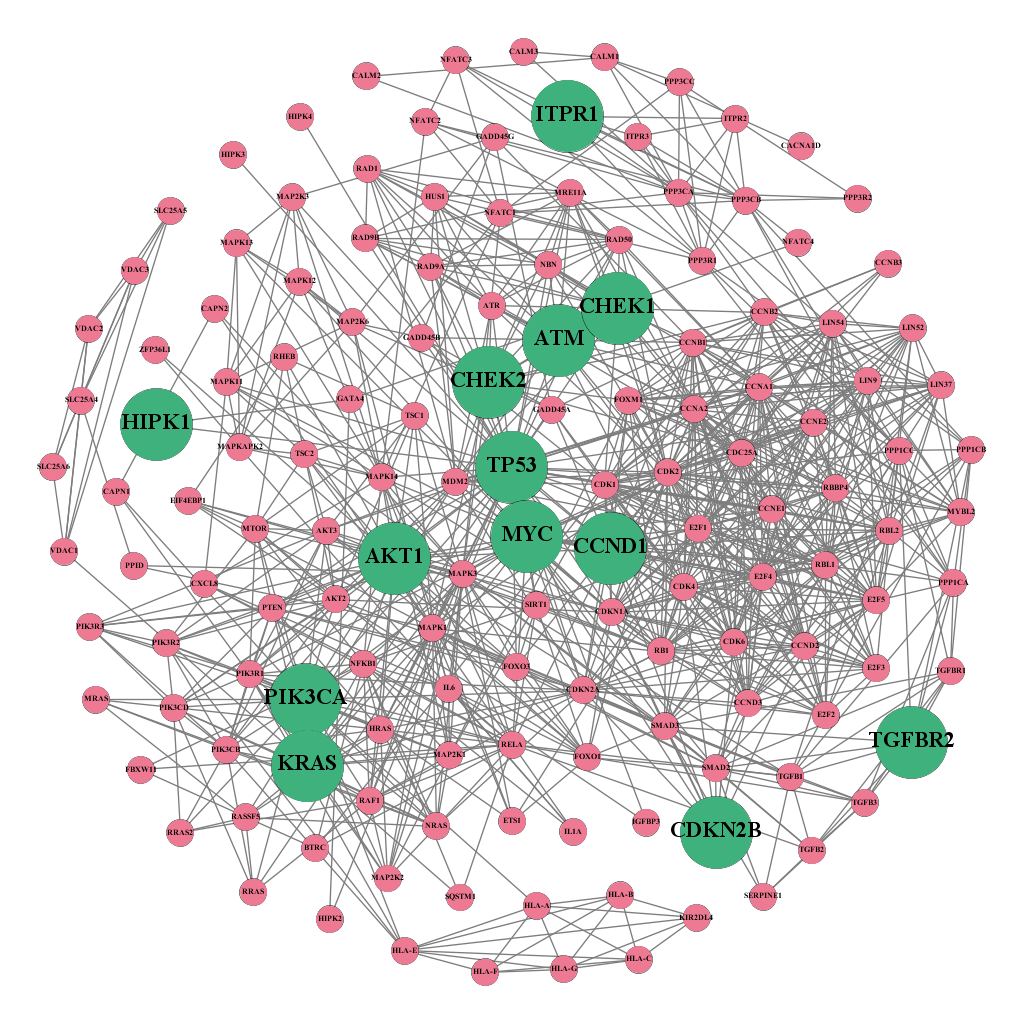


**Figure S4**. Subnetwork extracted from the PPI network by using the sets of genes of hsa04218: Cellular senescence.

**hsa05166 Human T-cell leukemia virus 1 infection**


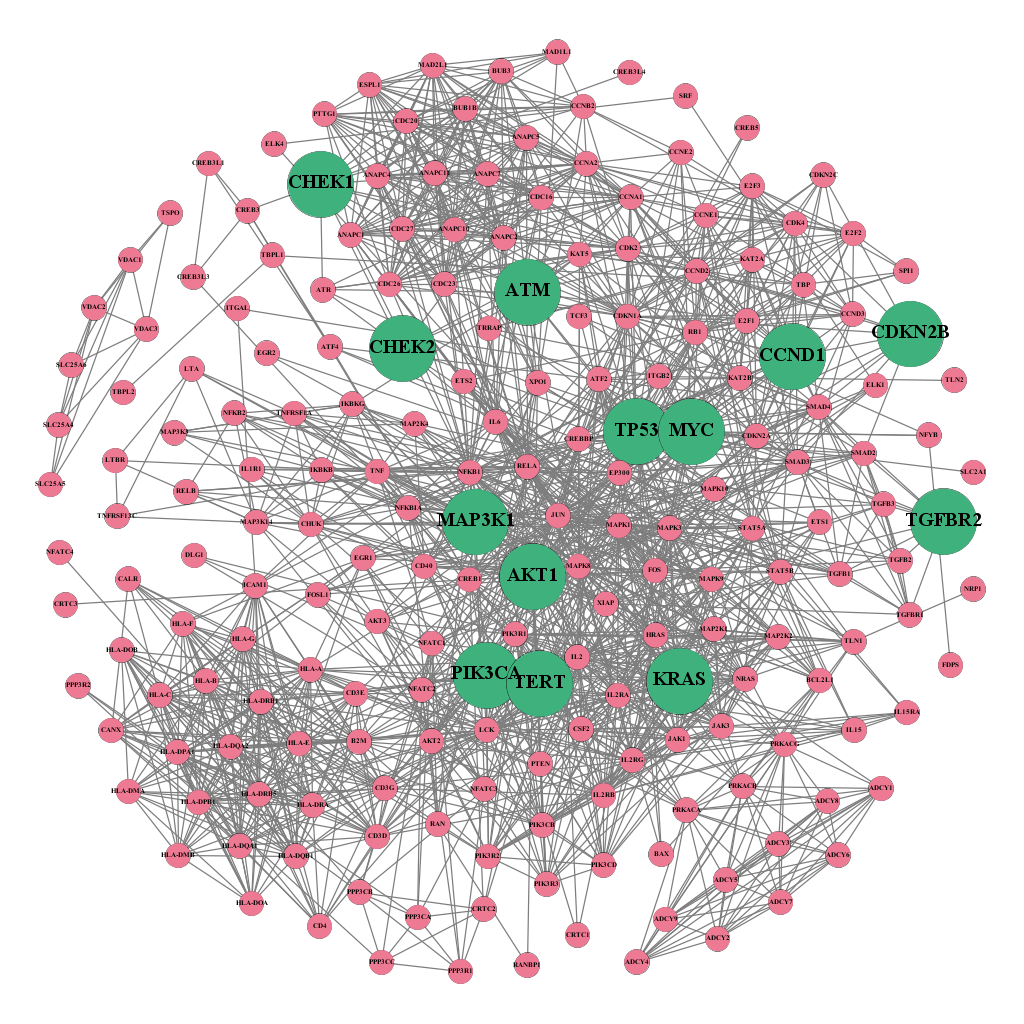


**Figure S5**. Subnetwork extracted from the PPI network by using the sets of genes of hsa05166: Human T-cell leukemia virus 1 infection.

**hsa05224 Breast cancer**


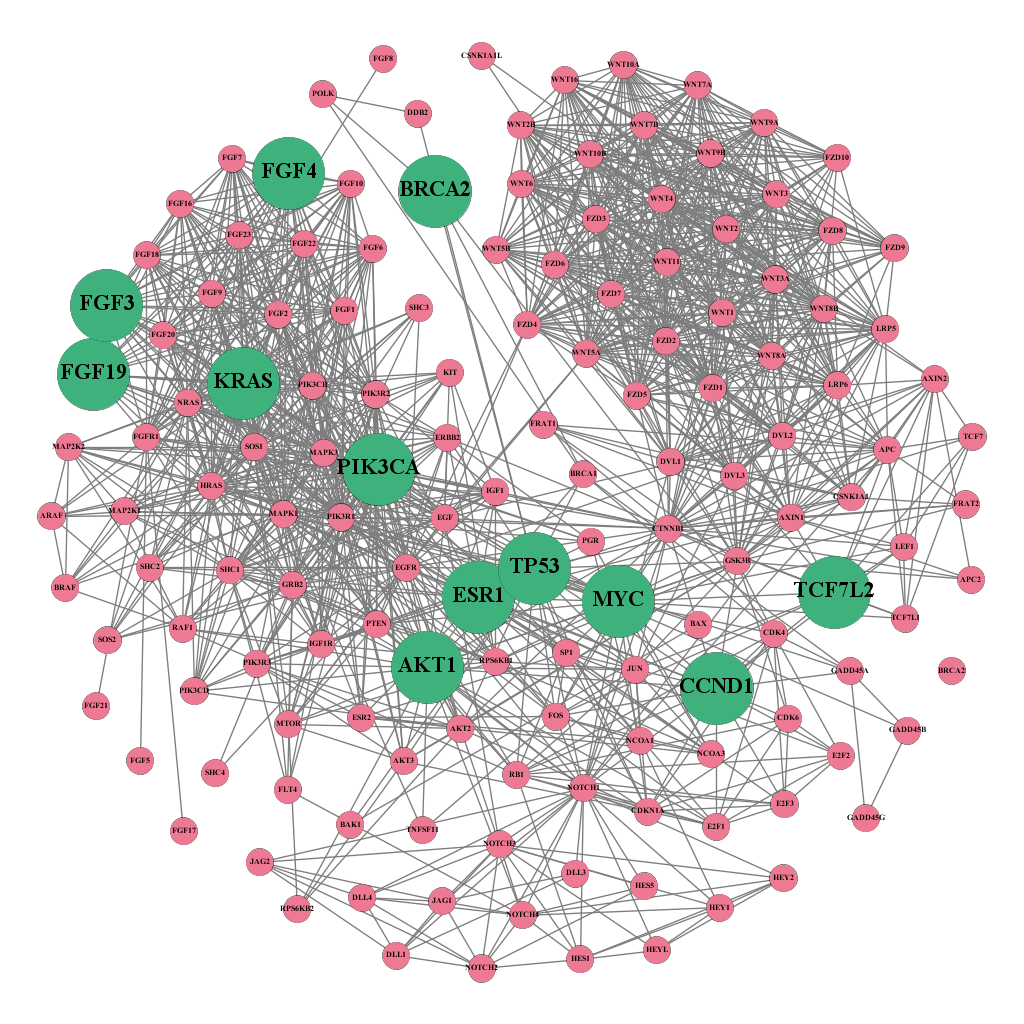


**Figure S6**. Subnetwork extracted from the PPI network by using the sets of genes of hsa05224: Breast cancer.

**hsa05218 Melanoma**


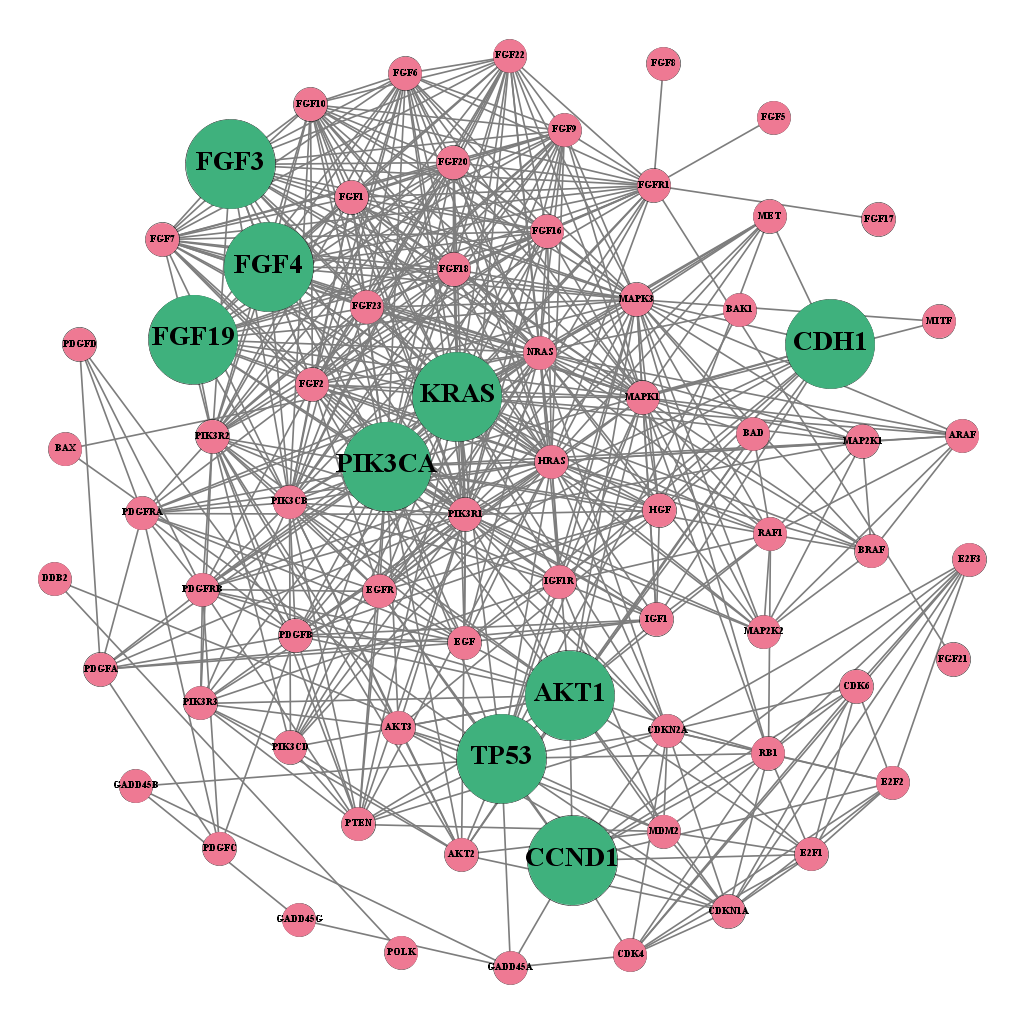


**Figure S7**. Subnetwork extracted from the PPI network by using the sets of genes of hsa05218 : Melanoma.


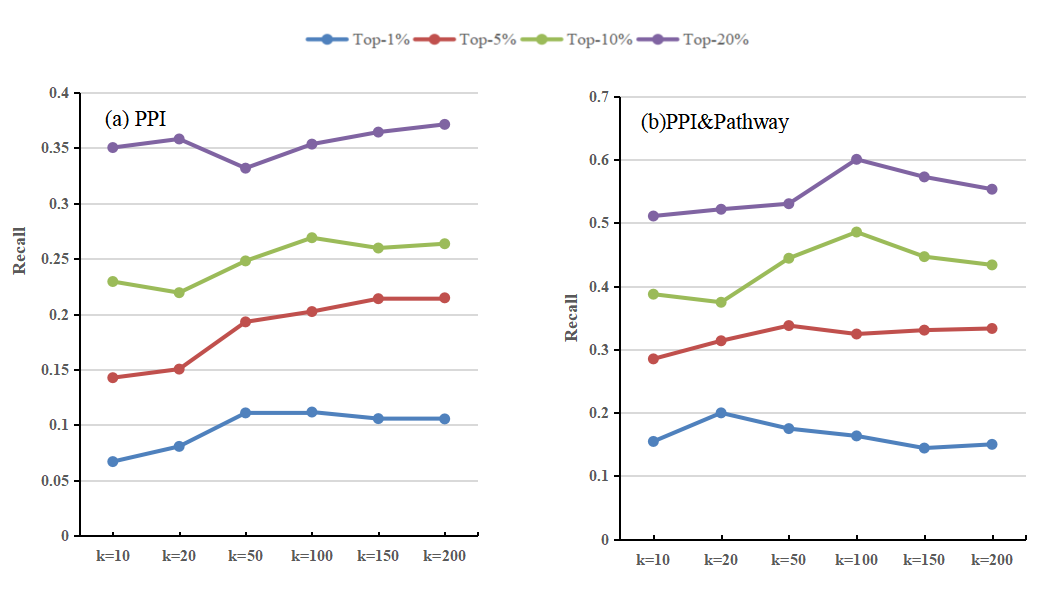


**Figure S8**.The performance of the RCRWR algorithm on (a) the PPI network and (b) the PPI network with KEGG pathway for top-1%,5%,10% and 20% when k value changes.

**[Appendix ] KEGG Pathway map**

**hsa05226 Gastric cancer**


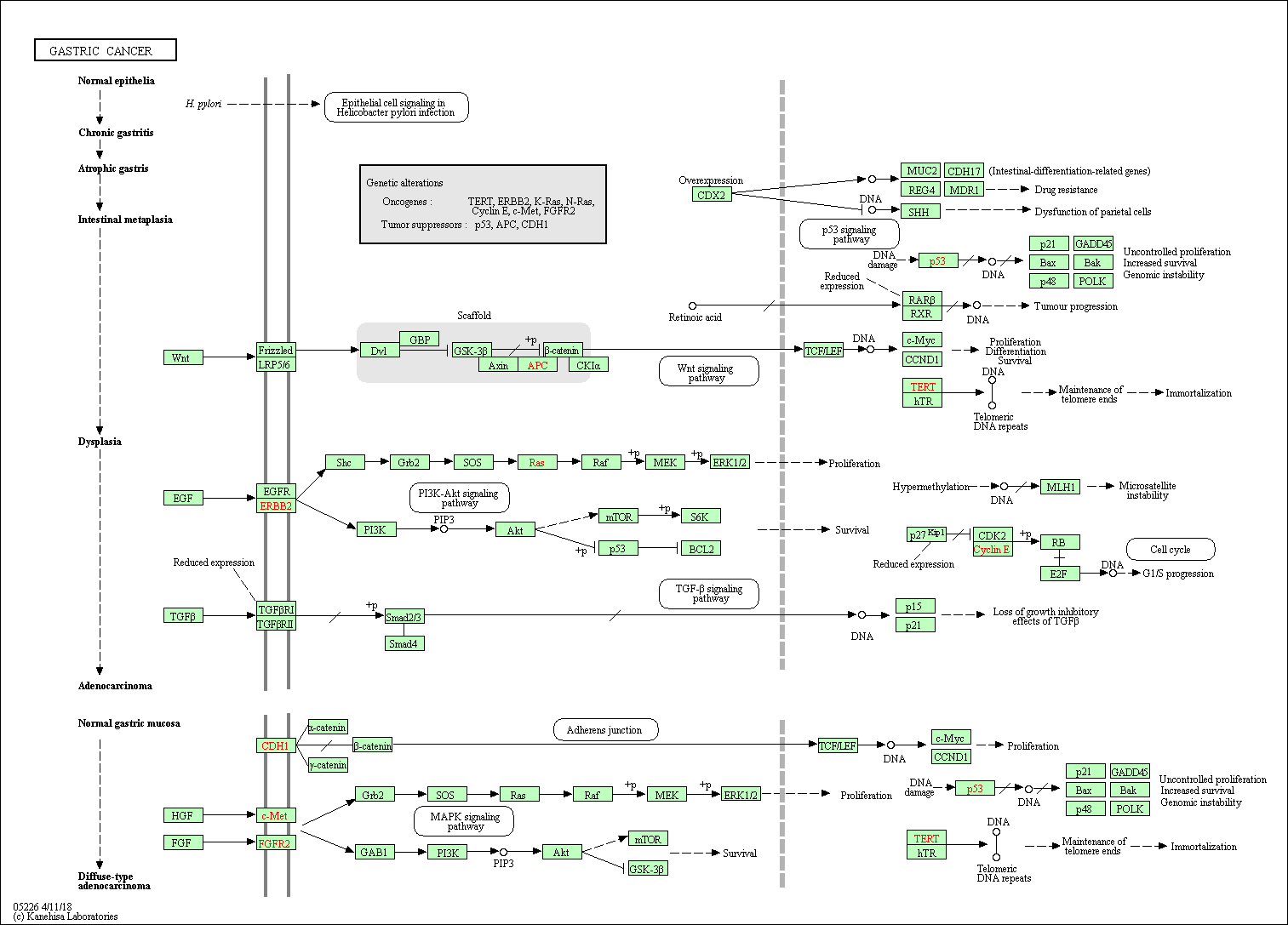


**Figure S9**. KEGG pathway map of hsa05226: Gastric cancer.

**hsa04218 Cellular senescence**


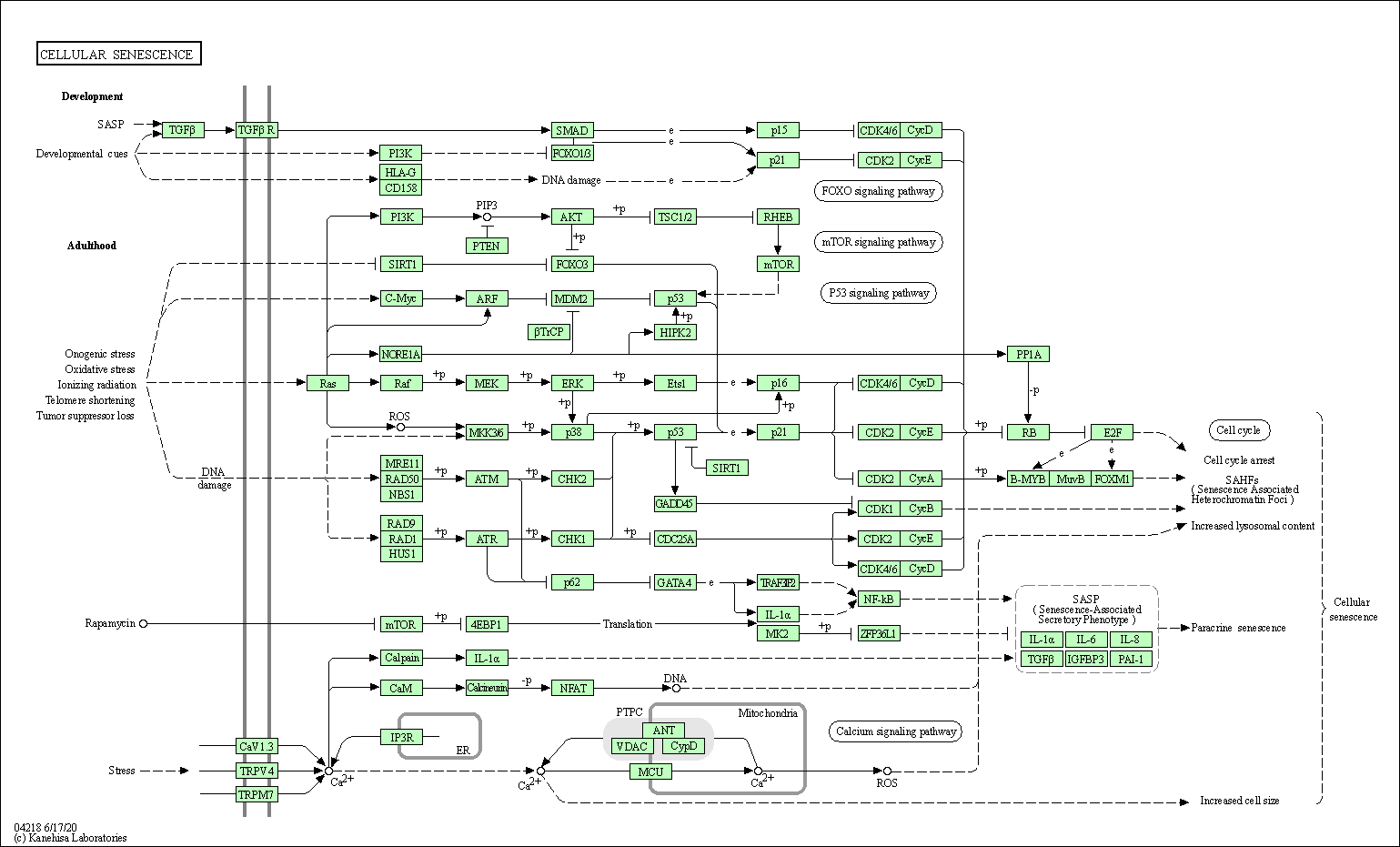


**Figure S10**. KEGG pathway map of hsa04218: Cellular senescence.

**hsa05166 Human T-cell leukemia virus 1 infection**


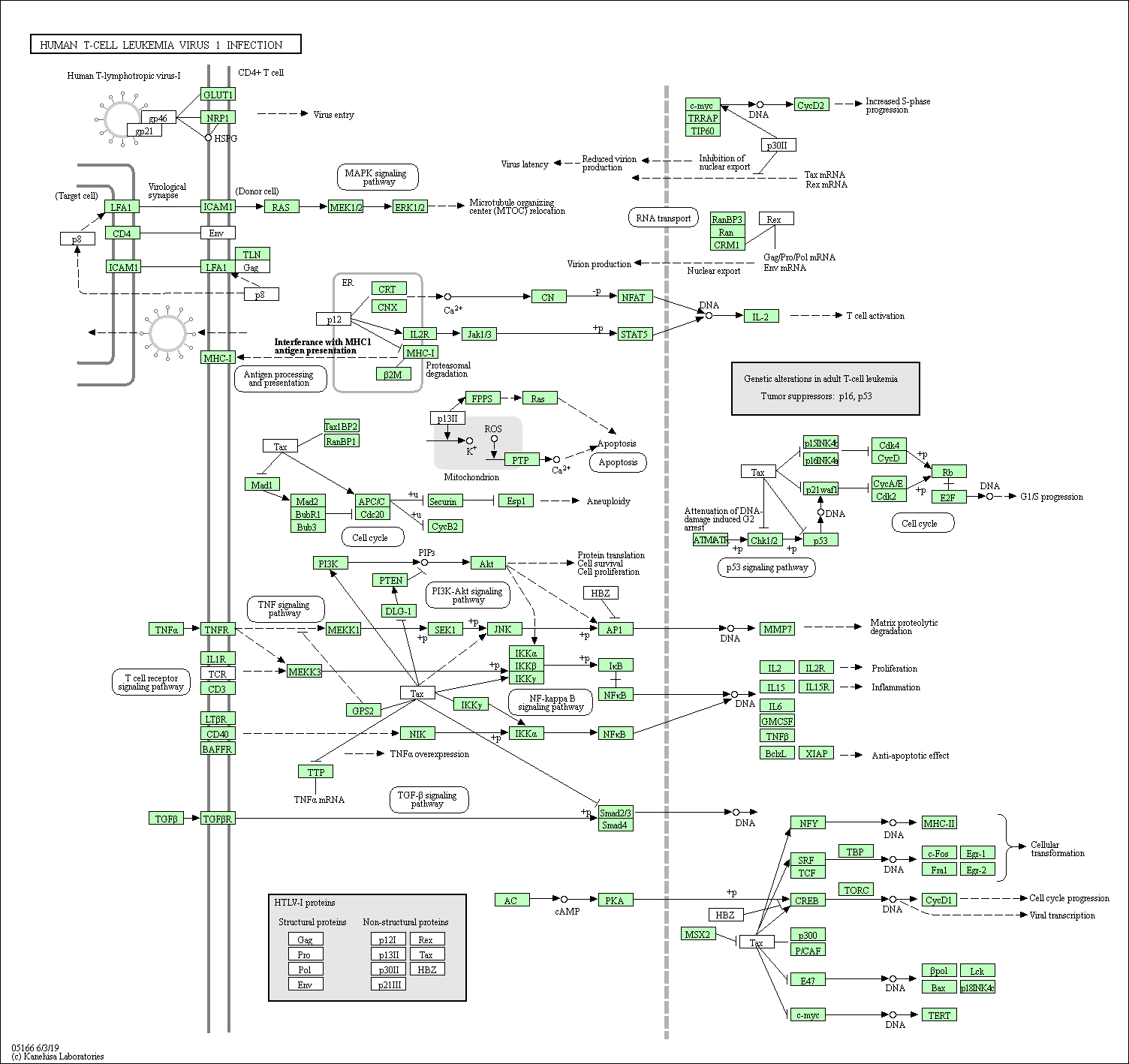


**Figure S11**. KEGG pathway map of hsa05166: Human T-cell leukemia virus 1 infection.

**hsa05224 Breast cancer**


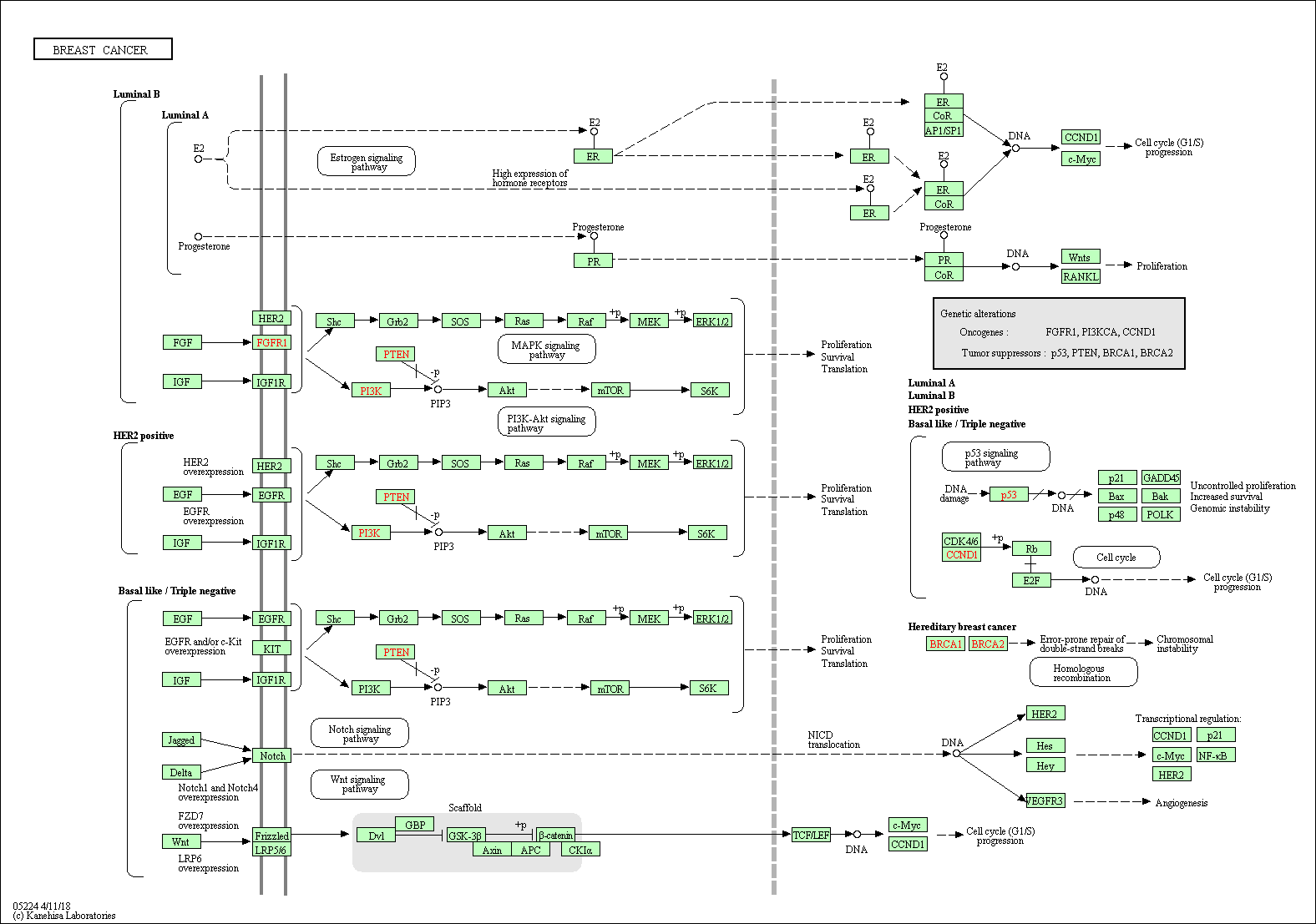


**Figure S12**. KEGG pathway map of hsa05224: Breast cancer.

**hsa05218 Melanoma**


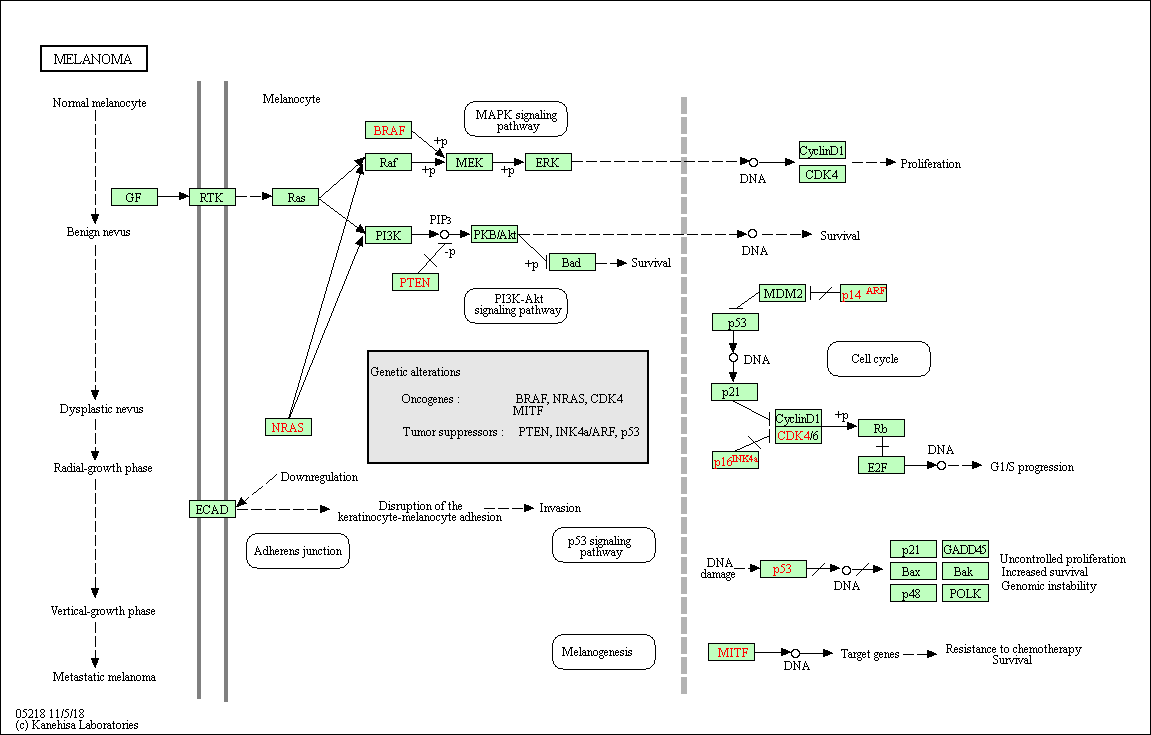


**Figure S13**. KEGG pathway map of hsa05218: Melanoma.
